# Efficient Aerial Water Harvesting with Self-Sensing Dynamic Janus Crystals

**DOI:** 10.1021/jacs.4c11689

**Published:** 2024-10-22

**Authors:** Linfeng Lan, Liang Li, Chenguang Wang, Panče Naumov, Hongyu Zhang

**Affiliations:** † State Key Laboratory of Supramolecular Structure and Materials, College of Chemistry, Jilin University, Changchun 130012, P. R. China; ‡ State Key Laboratory of Integrated Optoelectronics, College of Electronic Science and Engineering, Jilin University, Changchun 130012, P. R. China; § Smart Materials Lab, New York University Abu Dhabi, PO Box 129188, Abu Dhabi, UAE; ∥ Department of Sciences and Engineering Department, Sorbonne University Abu Dhabi, PO Box 38044, Abu Dhabi, UAE; ⊥ Center for Smart Engineering Materials, New York University Abu Dhabi, PO Box 129188, Abu Dhabi, UAE; # Research Center for Environment and Materials, Macedonian Academy of Sciences and Arts, Bul. Krste Misirkov 2, MK−1000 Skopje, Macedonia; g Molecular Design Institute, Department of Chemistry, New York University, 100 Washington Square East, New York, New York 10003, United States

## Abstract

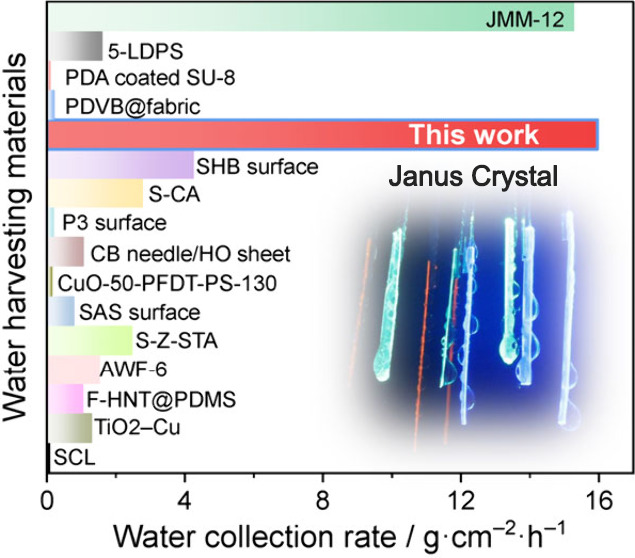

Water scarcity is one of the most pressing issues of contemporary societal development that requires innovative technologies where the material not only harvests water but also plays an active role in the process. Here, we demonstrate a highly efficient optical self-sensing approach to humidity capture from the air, where both humidity-harvesting and water-transduction functionalities are imparted on slender organic crystals by partial silanization via layer-by-layer hybridization. We report that due to the integration of the harvesting of aerial moisture and the collection of the condensed water, the ensuing Janus-type crystals capture humidity with the highest-to-date water collection efficiency of 15.96 ± 0.63 g cm^–2^ h^–1^. The water-collecting elements are also capable of delivering the water by reversible and periodic elastic deformation, and their high optical transparency allows real-time monitoring of the periodic fog collection process by deformational modulation of passively or actively transduced light that outcouples at the crystal-droplet interface. The results could inspire sophisticated approaches to humidity harvesting where optically transparent crystals combine fog capture with self-sensing capabilities for continuous and optimized operation to maximize the cost-gain balance of aerial fog capture.

## Introduction

With the escalating impact of global climate change and the increasing demand for renewables, optimizing the utilization of water resources is seen as paramount and urgent to prevent social injustice, political instability, and conflict.^1^ New techniques for water harvesting have become the primary goal of many research endeavors,^2,3^ with a particular focus on the development of new materials endowed with high water-harvesting capability.^4−6^ The Earth’s atmosphere contains an estimated 13,000 km^3^ of water,^7^ and harnessing this overlooked and underutilized resource places requirements on the material beyond the mere capability for adsorption and condensation of water. Materials with active functionalities, where the surface can adapt its water-collection propensity to the humidity level or other environmental effectors, can self-sense their operation, and even self-repair when damaged, are highly sought after. Our interest in exploring flexible crystalline materials for water harvesting stems from these materials’ unique structural characteristics and multifunctionality.^8−12^ Hybridization of flexible crystals with other material classes^25^ provides straightforward access to versatile sensing and actuating capabilities,^14,25^ cryogenic temperatures,^16^ magnetic fields,^17^ infrared light,^18^ and response to humidity.^20^ Here, we demonstrate that appropriately modified flexible organic crystals can be used to effectively harvest aerial humidity and collect the condensed liquid water, with simultaneous monitoring of the amount and motion of the water across the surface of the collector by capitalizing on the refractive index mismatch at the crystal-water interface. Unlike the previously reported porous organic crystals capable of easy and reversible adsorption of water at relative humidity (RH) levels above 55%,^26^ this is a new family of dual-functioning crystals that combine water-collecting and water-delivery functions entirely at their surfaces, realizing a new record in water collection efficiency at 95% RH. The action of the surface-modified crystals relies on consecutive capture of water vapor, transduction and delivery of the liquid water, and mechanical recovery to become available for cyclic operation, while their optical transparency offers means to monitor the process in real time optically. This latter effort highlights the ability of the crystals to outcouple passively or actively transduced light at their contact interface, thereby modulating the intensity of light signals. We also report that the water droplets can partially restore the optical transmission of partially fractured crystals by optical confinement of the light leakage at the fracture. This new approach is set to inspire a paradigm shift from static to *dynamic* humidity harvesting to optimize the process, minimize operational time, and maximize the amount of collected water.

## Results and Discussion

**Figure 1:**
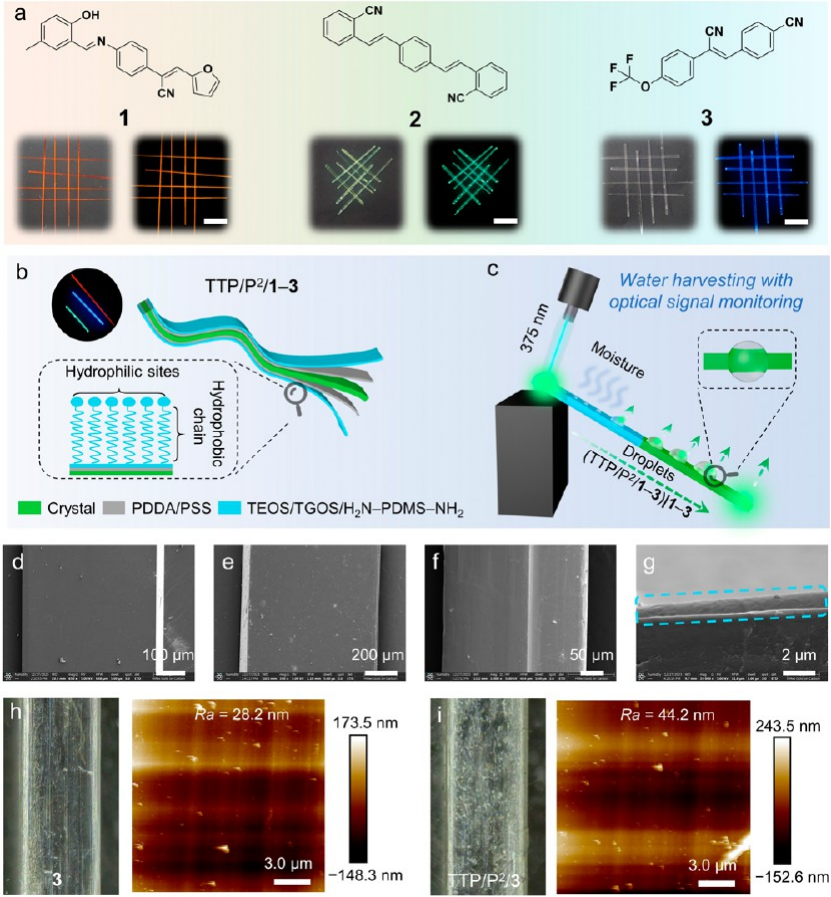
**Preparation and structure of the water-harvesting hybrid organic crystals**. (a) Chemical structures (top) and bright-field photographs (bottom) of typical crystals of **1**–**3** recorded under white light and ultraviolet light. The scale length is 5 mm. (b) A schematic showing the layered compositional profile of the hybrid crystals TTP/P^2^/**1**–**3**. (c) A schematic of the method for optical signal monitoring of the water harvesting with Janus crystals (TTP/P^2^/**1**–**3**)**|1**–**3**. As droplets are captured on the crystal surface and coalesce and slide down the crystal, the light outcouples at the crystal-water interface and results in modulation of the transmitted optical signal. (d–g) Scanning electron micrographs showing the surfaces of **3** (d), (PDDA/PSS)_5_/**3** (e), TTP/P^2^/**3** (f), and a cross-section of TTP/P^2^/**3** (g). The dashed line in panel g indicates the polymer layer on the crystal surface. (h, i) Optical micrographs and surface topologies, observed by atomic force microscopy, of crystals of **3** (h) and TTP/P^2^/**3** (i).

To demonstrate the generality of the approach, three chemically versatile organic compounds, **1**–**3** in Figure 1a^13,20^ were selected for their propensity to reproducibly grow as elastic crystals^8,27^ of up to centimeters in length. Although methods for the growth of crystals with controlled aspect ratio, such as the template-confined microcrystal growth method,^28,29^ are not readily applicable to preparing centimeter-scale crystals needed for water collection in this study, we were able to prepare crystals of varying sizes by adjusting the solution concentration in large containers, allowing us to select high-quality crystals with the desired dimensions. The width of most crystals was a few hundred microns; however, several lamellar crystals of **2** wider than 5 mm were also obtained. The surfaces of the latter allowed for static contact angle (θ_SCA_) measurements and were found to be weakly hydrophilic (θ_SCA_ = 70.5 ± 0.9° for pristine **2**; Supplementary Figure 1). Suitably long acicular crystals of **1**–**3** were layer-by-layer hybridized over five reiterations with alternating oppositely charged polyelectrolytes poly(diallyldimethylammonium chloride) (PDDA) and poly(styrene sulfonic acid) sodium (PSS),^30^ rendering their surfaces adhesive and hydrophilic (θ_SCA_ = 46.9 ± 0.8° for (PDDA/PSS)_5_/**2**). The coated crystals were then silanized with a mixture of tetraethylorthosilicate (TEOS), triethoxy(3-glycidyloxypropyl)silane (TGOS), and *bis*(3-aminopropyl)-terminated poly(dimethylsiloxane) (H_2_N–PDMS–NH_2_), fumed over acid for hydrolysis and condensation, and dried (Supplementary Figure 2).^31,32^ This protocol afforded hybrid organic crystals TTP/P^2^/**1**–**3** having approximately 886 ± 66 nm thick TTP/P^2^ polymer coating (Figure 1b) that imparts surface hydrophobicity (θ_SCA_ = 96.4 ± 0.8° for TTP/P^2^/**2**). At a microscale, the surface of the hybrid crystals appeared smooth and uniform (Figure 1d–g; Supplementary Figure 3), while at a nanoscale, it was significantly rougher relative to the pristine crystals, with the average surface roughness over a 250 μm^2^-sampling area increasing from *Ra* = 28.2 to 44.2 nm (Figure 1h, i). Similar to the native crystals, the hybrid crystals TTP/P^2^/**1**–**3** had good elasticity and could be bent strongly and repeatedly without fracturing (Supplementary Figure 4). The polymer layer was optically transparent, reducing the fluorescence of the original uncoated crystal by only about 5% (Supplementary Figure 5), and had a marginal effect on their flexural and fracture strength with only a slight reduction in stiffness (Supplementary Table 1, Supplementary Figure 6).

**Figure 2:**
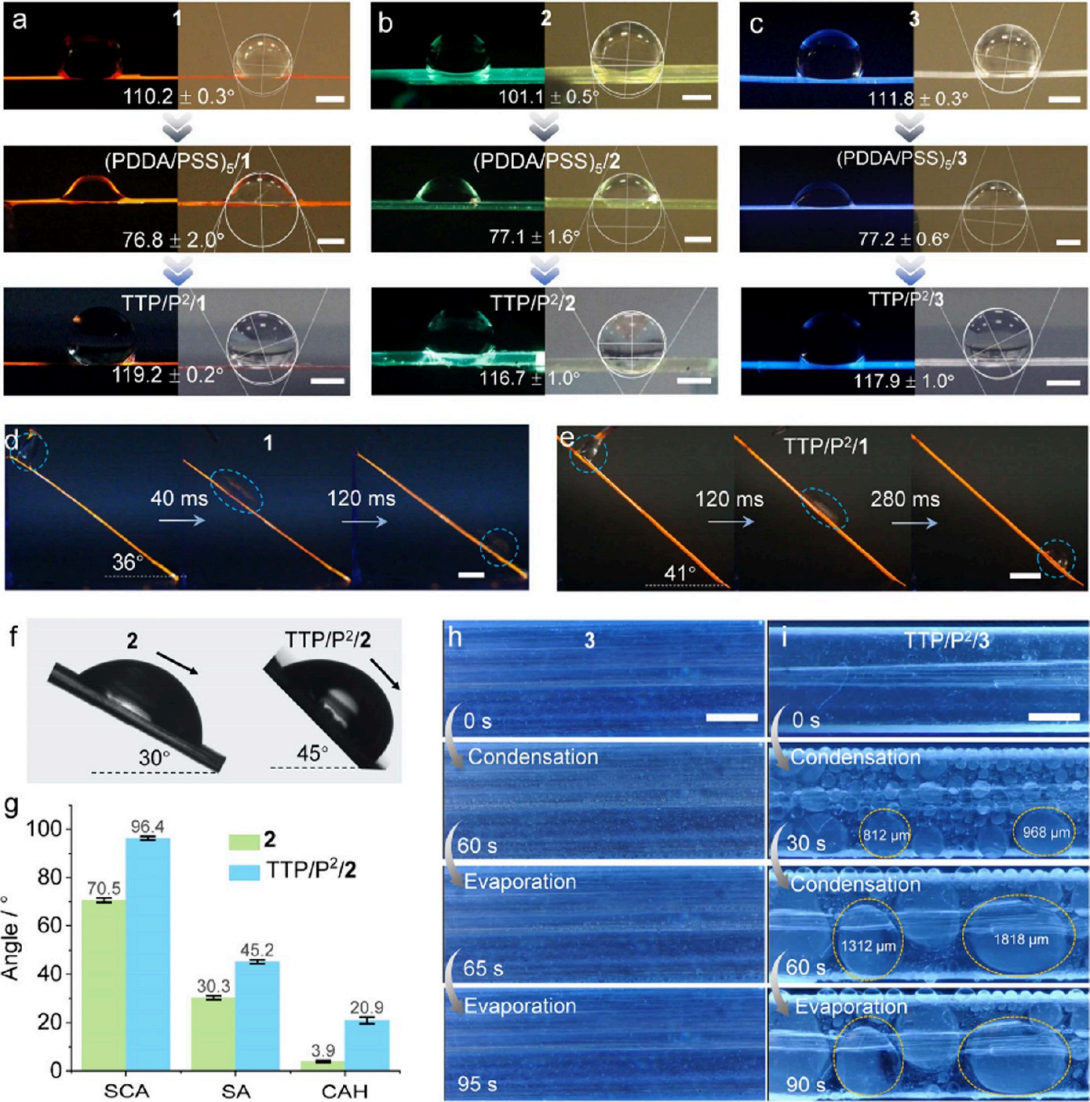
**Surface properties and wettability of the hybrid crystals.** (a–c) Photographs of water droplets (4 μL) sitting on the surfaces of crystals observed under 365 nm UV light (left; UV light was used for enhanced contrast between the fluorescent crystals and the dark background) and daylight (right). The angles in panels a–c correspond to the attachment angles (θ_AA_) measured when the droplet size is larger than the crystal width (note that these values are different from the contact angles). (d, e) Sliding of water droplets (4 μL) across the surface of inclined crystals of **1** and TTP/P^2^/**1**. (f) A 20-μL droplet sliding down crystals of **2** and TTP/P^2^/**2**. (g) Comparison of the static contact angle (θ_SCA_), sliding angle (θ_SA_), and contact angle hysteresis (θ_CAH_) between crystals of uncoated **2** and TTP/P^2^/**2**. (h, i) Water capture and evaporation occurring at the surfaces of **3** (h) and TTP/P^2^/**3** (i) recorded at room temperature under UV light. The scale length in panels a–c, h, and i is 1 mm, and in panels d and e it is 2 mm.

With the crystals **1**–**3** being only tens to thousands of microns wide, a microliter water droplet placed on the crystal was larger than the width of the crystal. However, the weakly hydrophobic surface rendered the crystal moderately adhesive to water; in effect, the water droplet typically engulfed a horizontally positioned crystal and remained attached to it (Figure 2a–c). The contact boundary effect between the droplet and the crystal caused the actual attachment angle (θ_AA_), which is conducive to water motion across the surface, to be greater than the measured contact angle (θ_CA_). As a result, when the crystal was tilted, the water droplet slid rapidly down its longest axis (Supplementary Movie 1). For instance, a 4 μL-droplet, having a diameter *d* ≈ 2 mm, placed on a 36°-tilted pristine crystal of **1** traveled 11.5 mm in only 120 ms (0.095 m s^–1^; Figure 2d), while a water droplet on TTP/P^2^/**1** slid down the 41°-tilted crystal over only 5.2 mm for the same time interval (0.043 m s^–1^; Figure 2e). The result indicates greater potential for rapid transport of water by the uncoated crystals and superior performance in water transport, even though they are more wettable compared to the coated surface. The dynamic contact angles were determined on wider lamellar crystals with widths >5 mm that were available only with **2** and TTP/P^2^/**2**. The droplets (20 μL) on the pristine crystal started to slide at a smaller sliding angle (θ_SA_) compared to the coated crystal (θ_SA_ = 30.3 ± 0.8° vs 45.2 ± 0.8°) and had much smaller contact angle hysteresis (θ_CAH_), θ_CAH_ = 3.9 ± 0.4° vs 20.9 ± 1.3° (Figure 2f, g; Supplementary Figure 7). The smaller θ_SA_ and θ_CAH_ of the original crystal confirmed the more efficient transduction of liquid water by the uncoated crystals relative to the polymer-coated crystals.

**Figure 3:**
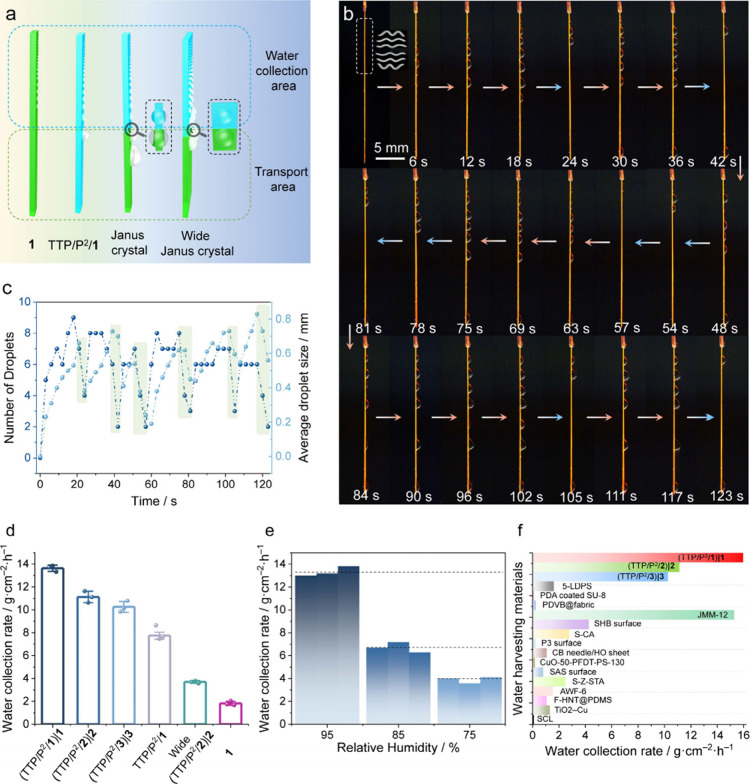
**Fog collection performance.** (a) A schematic showing the humidity harvesting by using an original (uncoated) crystal of **1**, a fully coated crystal TTP/P^2^/**1**, Janus crystals (TTP/P^2^/**1**–**3**)**|1**–**3**, and the wide lamellar Janus crystal (TTP/P^2^/**2**)**|2**. (b) Snapshots showing the fog collection process of a fully coated crystal TTP/P^2^/**1** within 123 s. The distance between the sample and the source of airflow (0.25 L h^–1^) was kept at 5 cm. (c) Periodic changes in the number and average size of droplets during fog collection of TTP/P^2^/**1**. (d) Water collection rates of (TTP/P^2^/**1**–**3**)**|1**–**3**, all-coated TTP/P^2^/**1**, wide lamellar (TTP/P^2^/**2**)**|2**, and the original, uncoated crystal of **1**. (e) Water collection rates of (TTP/P^2^/**1**)**|1** at 95%, 85%, and 75% RH (each experiment was performed in triplicate). (f) Comparison of the water collection rates of the Janus crystal (TTP/P^2^/**1**–**3**)**|1**–**3** (the values are listed in Supplementary Table 2) with other water-harvesting materials reported earlier (the literature values were determined between 80–95% RH).^36−50^

When placed in very moist air with 95% relative humidity (RH), water droplets were observed to condense on >2 mm-wide crystals of both uncoated **2** and **3** and on the coated crystals TTP/P^2^/**2** and TTP/P^2^/**3**, however, with very different patterns (Figure 2h, i; Supplementary Movies 2 and 3). For crystals of uncoated **2** and **3**, multiple droplets with a diameter *d* = 50–100 μm appeared on their surface and started to coalesce after about 10 s (Figure 2h; Supplementary Figure 8). After 60 s, the droplets increased in size only marginally and did not continue to aggregate into larger droplets. Upon transfer of the crystals to air with 20% RH, the droplets evaporated in less than 60 s (Figure 2h; Supplementary Figure 9), indicating that the nascent crystals cannot retain water. In contrast, the coated crystals TTP/P^2^/**2** and TTP/P^2^/**3** condensed a higher density of larger droplets, with *d* = 100–220 μm (Figure 2i; Supplementary Figure 10). Over time, the droplets increased in size and, after 60 s, started to coalesce into even larger droplets, with *d* > 1 mm. In contrast with the uncoated crystals, at 20% RH, these droplets remained on the surface. The retention, growth, and coalescence of droplets are attributed to the presence of both hydrophilic and hydrophobic functional groups in the coating, among other factors that could synergistically impart water-collection capability.^32^ To test the fog collection under dynamic conditions, a crystal of TTP/P^2^/**1** was positioned vertically, a stream of moist air was applied locally to an area of approximately 0.06 cm^2^, and the condensation was observed under UV light for enhanced contrast against a black background (Figure 3a, b). At 24 s, three droplets of condensed water slid off the crystal surface into a receptacle, and by 57 s, the surface of the crystal was devoid of liquid water, indicating that equilibrium has been reached. The droplet growth and sliding occurred periodically when their number and size reached a threshold, and the process proceeded cyclically (Figure 3c).

The very different surface properties between the coated and uncoated sections of the crystal inspired us to prepare crystals that are half-coated along their longest axis (Figure 1c, Figure 3a), for which we propose the term “Janus crystals” in analogy to other objects (for example, nanoparticles) that have opposite faces with distinct physicochemical properties.^33−35^ Unlike the nascent crystals and the completely coated crystals, the specific composition of materials in the Janus crystals brings about a unique combination of features: (1) a polymer layer containing hydrophilic amino groups for water capture and condensation, coupled with hydrophobic silane chains that promote droplet accumulation; (2) narrow crystals that facilitate the migration of droplets with optical transparency for optical monitoring; and (3) a reduced sliding angle and contact angle hysteresis in the crystal layer that facilitates the transport of liquid water for continued collection. In the Janus crystal (TTP/P^2^/**2**)**|2**, for example, the coated sector (TTP/P^2^/**2**) condenses the water droplets and facilitates their coalescence into larger droplets. After the droplets have reached a critical size, they slide down the crystal under gravity to its uncoated sector (**2**), which then facilitates the transport of the water to the bottom terminus of the crystal, where it is collected.

The water-collecting performance was assessed by using a customized fog-harvesting system (Figure 3; Supplementary Movies 4–6 and Supplementary Table 2). The water collection rates of the Janus crystals (TTP/P^2^/**1**–**3**)**|1**–**3** were determined to be 13.63 ± 0.27, 11.13 ± 0.51, and 10.26 ± 0.47 g cm^–2^ h^–1^, respectively. The wide lamellar Janus crystals (TTP/P^2^/**2**)**|2** showed a smaller rate of 3.96 ± 0.11 g cm^–2^ h^–1^ (Figure 3d). These values represent a drastic improvement compared to uncoated **1**, which collected water at a rate of 1.82 ± 0.17, and TTP/P^2^/**1**, at 7.75 ± 0.31 g cm^–2^ h^–1^ (Supplementary Table 2). The effect of humidity level was further studied on the best-in-class Janus crystal, (TTP/P^2^/**1**)**|1** (Figure 3e, Supplementary Table 3). In three parallel experiments, the water collection efficiency of this material was 13.33 ± 0.43 g cm^–2^ h^–1^ at ∼95% RH. At lower humidity of ∼85% and ∼75% RH, the water collection efficiency was reduced to 6.71 ± 0.44 and 3.90 ± 0.29 g cm^–2^ h^–1^, respectively. Based on the low water collection rate of lamellar Janus crystals, we also investigated the effect of the crystal width on the water harvesting efficiency. We observed that as the width of the Janus crystals (TTP/P^2^/**1**)**|1** decreased from 452 to 150 μm, the water collection rate increased from 10.27 ± 0.22 to 15.96 ± 0.63 g cm^–2^ h^–1^ (Supplementary Table 4). However, it is worth noting that when the crystals become too small and narrow, it becomes more challenging to modify their surface, which ultimately has a negative effect on their water collection efficiency (Supplementary Figure 11). In addition, the water collection performance of Janus crystals (TTP/P^2^/**1**–**3**)**|1**–**3** with similar dimensions showed no significant difference, indicating that molecular structure does not affect their water collection efficiency (Supplementary Figure 12, Supplementary Table 5).

As shown in Figure 3f, the value of 15.96 ± 0.63 g cm^–2^ h^–1^ of (TTP/P^2^/**1**)**|1** is the highest among the reported water-harvesting materials (note that the literature values were determined between 80–95% RH),^36−50^ surpassing all previously humidity-collecting materials and is comparable to the current record-holding material, a mesh membrane reported to collect fog at a rate of 15.28 g cm^–2^ h^–1^.^37^ We note that, unlike some other water-collecting materials and devices, the Janus crystals described here carry the advantage of simple preparation, and they provide space to explore chemical versatility for optimized efficiency (Figure 3f, Supplementary Table 6).^36−50^ The capability for cyclic operation of the water collection system is critically important from the view of its practical application. In order to study the performance over prolonged periods of operation, Janus crystal (TTP/P^2^/**1**)**|1** was exposed to 95% RH for 6 h daily over 6 days and its water collection efficiency was monitored. As shown in Supplementary Figure 13 and Table 7, the crystal maintained high efficiency after prolonged use, demonstrating exceptional durability.

**Figure 4:**
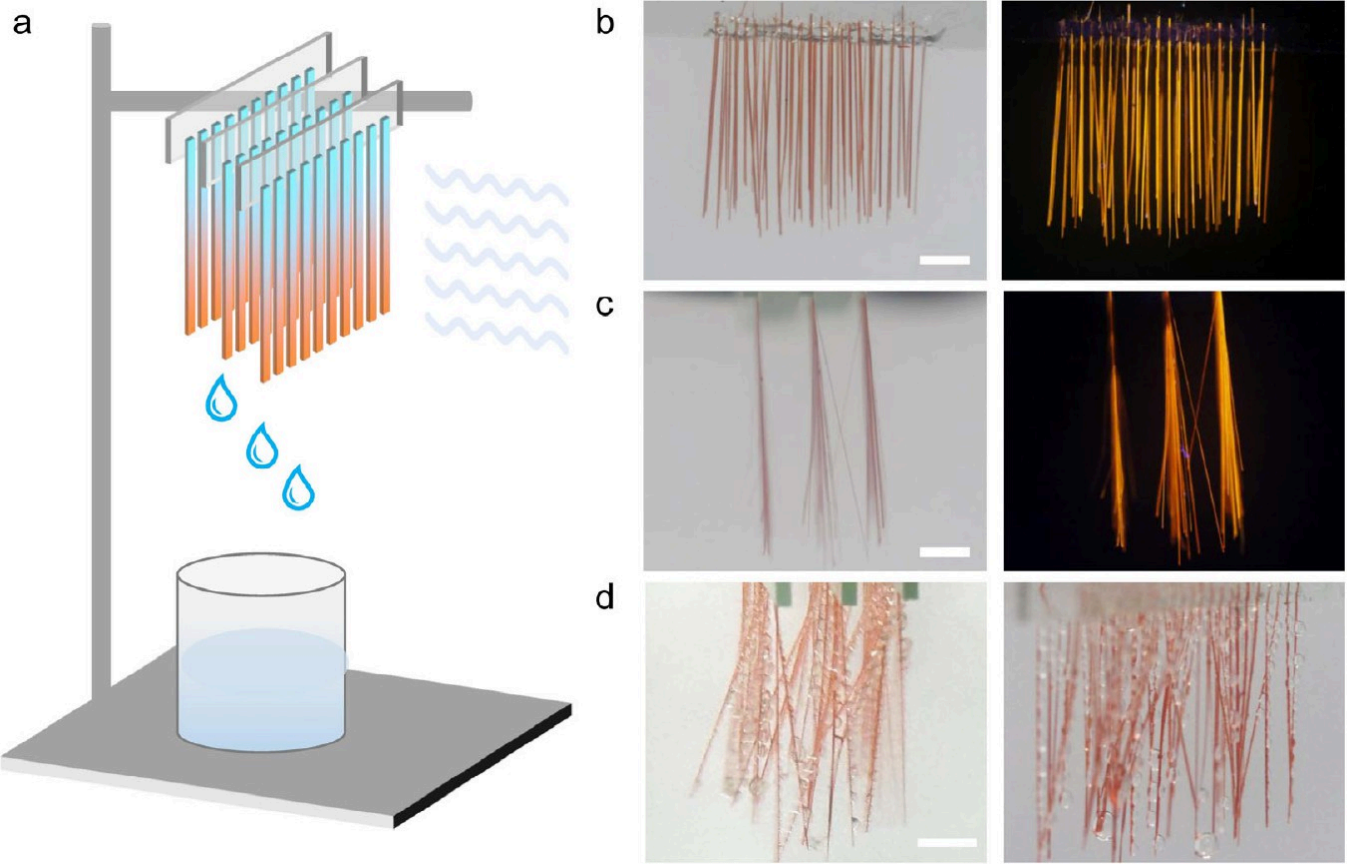
**Collective water collection.** (a) Schematic of the setup for humidity harvesting by using a collection of crystals. The distance between the samples and the source of airflow (∼0.15 L h^–1^) was kept at 9–10 cm. (b, c) Frontal (b) and lateral (c) views of bundles of Janus crystals TTP/P^2^/**1** in daylight and under UV light. (d) Snapshot of the Janus crystals bundles as they collect water. The scale length in all panels is 5 mm.

To increase the efficiency, as well as to explore the possible collective effects in humidity harvesting, we aimed at increasing the active area for water condensation by crystal bundling. Sixty Janus crystals (TTP/P^2^/**1**)**|1** with widths 107–325 μm (Supplementary Table 8) were affixed on a glass sheet, binned into three bundles of 20 crystals each, and spaced about 3 mm apart (Figure 4a–c, Supplementary Figure 14). When exposed to an environment of about ∼85% RH, the crystal surfaces were covered with droplets in a few minutes (Figure 4d). After collecting water for 8 h, the Janus crystals with a total area of about 1.76 cm^2^ collected 42.679 g of water (Supplementary Movie 7), with an efficiency of 3.03 g cm^–2^ h^–1^. The decline in water collection efficiency relative to individual crystals is attributed to the fact that in the process of collective water collection, the larger droplets occasionally cause the crystals to stick together, thereby reducing the droplet flow. Infrared spectra of water collected by the Janus crystals (TTP/P^2^/**1**–**3**)**|1**–**3** and deionized water (Supplementary Figure 15) confirmed the lack of contamination of the water with the organic compounds or the polymers. Moreover, fog collection tests performed outdoors using nine Janus crystals (TTP/P^2^/**1**–**3)|1**–**3** showed that, due to the influence of wind and droplet evaporation, only 0.252 g of water could be collected over 8 h (from 9 p.m. to 5 a.m.; Supplementary Figure 16). This result highlights the need for design of a water-collection device to enhance the retention of the captured water for practical applications.

**Figure 5:**
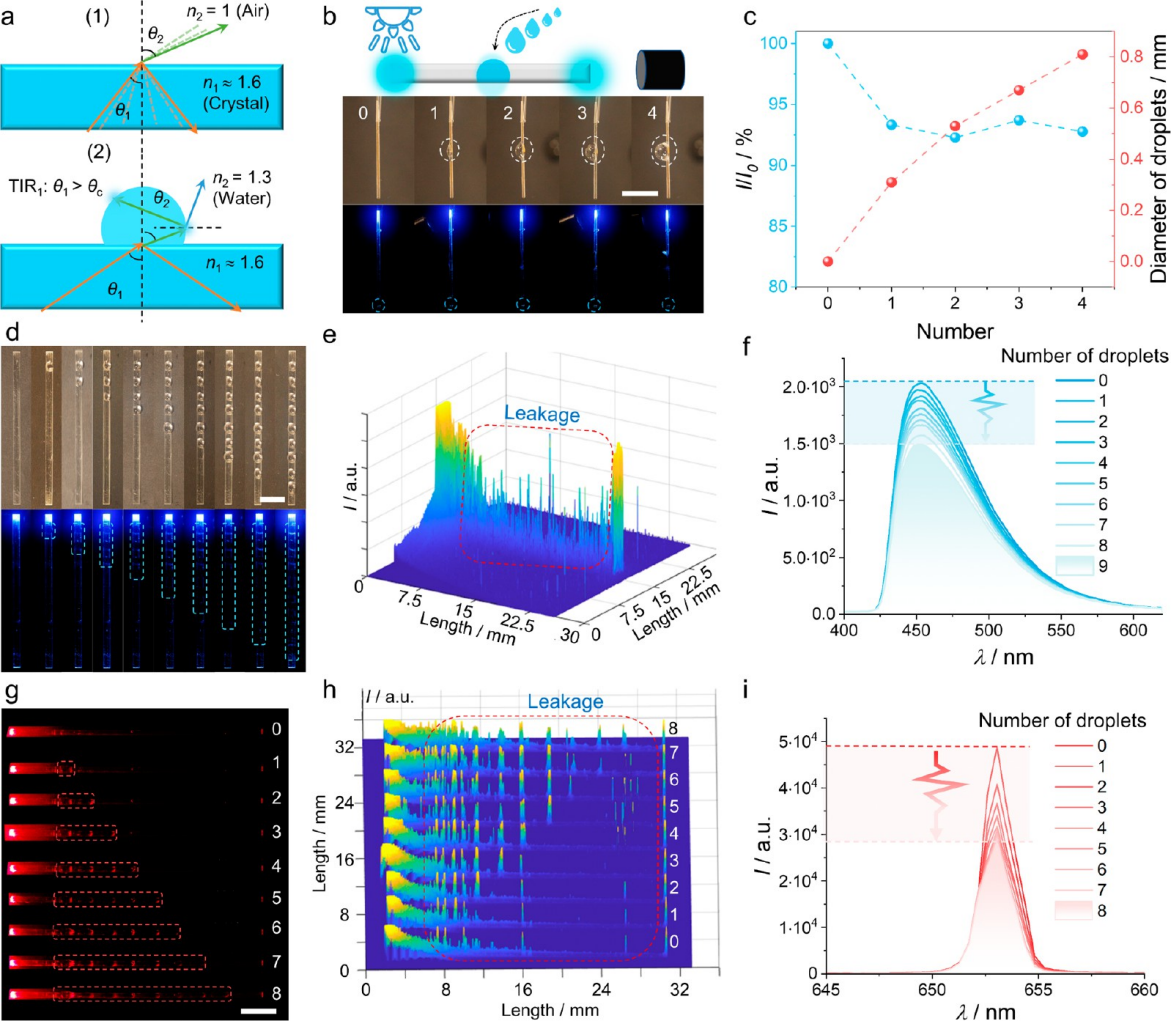
**Optical sensing of the water collection process.** (a) Snell’s refraction condition applied to an organic crystal without and with a droplet of water on its surface. (b) Effect of the droplet size on the transmission of light through the crystal, and photographs of droplets of different sizes on the surface of a crystal of TTP/P^2^/**3**. (c) Relationship between the size of a single droplet and the optical output signal delay. (d) Effect of the number of water droplets placed on the surface of TTP/P^2^/**3** on the light output. The crystal was excited with a 355 nm laser. (e) Three-dimensional (3D) light intensity map of a crystal of TTP/P^2^/**3** carrying eight droplets. (f) Output spectra corresponding to the transmission of the crystal shown in panel e. (g) Passive optical waveguiding by a crystal of TTP/P^2^/**3** having different numbers of droplets on its surface. The crystal was excited with a 654 nm laser. (h, i) 3D light intensity maps and corresponding spectra showing output signal changes of TTP/P^2^/**3** carrying a different number of droplets. The scale length in panel b is 2 mm, and in panels d and g, it is 4 mm.

Considering that the coated crystals remain optically transmissive, we further studied the effect of water condensation on the optical waveguide profile of the crystals. The total internal reflection of light within the crystal requires that the refractive index of the crystal be higher than that of the surrounding medium and the incident angle be greater than the critical angle (θ_c_).^12^ For a dry crystal of TTP/P^2^/**3** with a refractive index *n* ≈ 1.6 in air (*n* = 1.0), the internal reflection occurs from the crystal’s internal surface above the critical angle θ_c_ ≈ 38.7° and is contained within the crystal, which acts as an active optical waveguide^51^ (Figure 5a; for details on the waveguide characterization, see the Methods section). A droplet of water (*n* = 1.3, 4 μL) on the surface decreases the critical angle to θ_c_ ≈ 56.2°, resulting in partial outcoupling of the light at the crystal-water interface and increased optical loss. The light leakage at the crystal-droplet contact interface caused the intensity of the emission signal at the terminal to drop to about 93% of that of the original signal (Supplementary Figure 17).

The effect of the amount and distribution of condensed water on the fluorescence transmission through the crystal was further explored by varying the size and number of droplets on a crystal of TTP/P^2^/**3** excited at 355 nm (Figure 5b, c). With a single droplet placed in the middle of the crystal, the signal intensity of the 453 nm emission from the crystal, which acts as an active optical waveguide, was essentially unaffected by the droplet size (Figure 5c). Since each droplet acted as a light-dissipating gate, however, increasing the number of droplets decreased the signal intensity. Stepwise application from one to nine nearly equidistant (2.5 mm) droplets of diameter ∼1.2 mm on TTP/P^2^/**3** decreased the emission intensity (*I*) incrementally relative to the value of the dry crystal (*I*_0_, Figure 5d–f), resulting in an intensity ratio *I*/*I*_0_ = 73% when all nine droplets were applied. The linear correlation between *I*/*I*_0_ and the number of droplets (Supplementary Figure 18) indicates that the output light intensity can be used to monitor the water collection process. Given that the optical absorption of water is wavelength-dependent,^52^ the sensing capability was also tested for passive (non-emissive) optical transduction of light through the crystal at 654 nm (Figure 5g). The light output of the red light was found to decrease even more substantially (Figure 5h, i), with *I*/*I*_0_ = 56% for eight droplets (Supplementary Figure 19).

**Figure 6:**
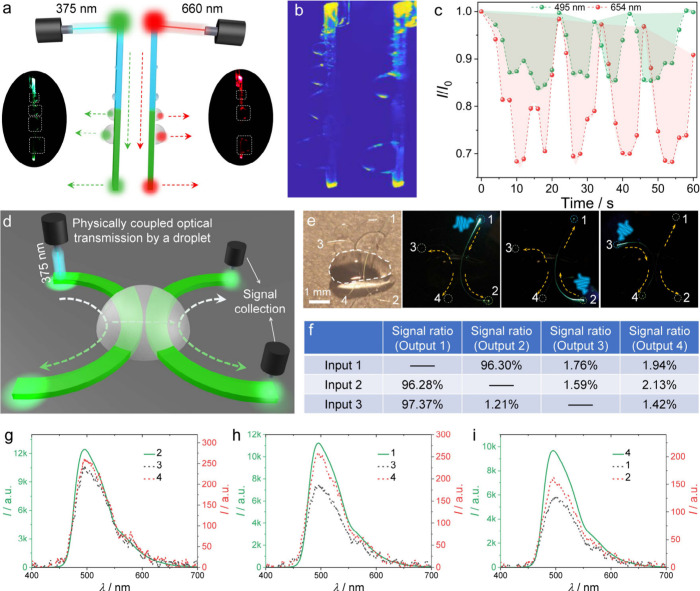
**Optical monitoring and coupling of the water collection process.** (a) Optical signal monitoring of water harvesting by a hybrid crystal in active and passive modes. The dotted arrows indicate the direction of optical signal transmission. The insets are photos of active and passive optical waveguides with droplets. (b) Two-dimensional (2D) light intensity maps of crystals of (TTP/P^2^/**2**)**|2** acting as active (left) and passive (right) waveguides during water harvesting. (c) Variation of the passively and actively transduced signal ratio (*I*/*I*_0_) with time during the fog collection. The shaded area reflects the periodic active state of the fog collection process. (d) A schematic of a 2 × 2 waveguide coupler. Two curved crystal waveguides are coupled through a water droplet. (e) Photographs of the coupler and the optical coupling as different terminals were excited. (f) Signal ratio when terminals 1–3 are excited. (g–i) Emission spectra recorded when terminals 1 (g), 2 (h) and 3 (i) are excited.

The optically transparent, uncoated, and partially or fully coated crystals allow real-time monitoring of fog collection through optical transduction (Figure 6a). Water coalescence and evolution on the crystal surface significantly impacts the optical waveguide signal. A 1 cm-long Janus crystal (TTP/P^2^/**2**)|**2** with a 5 mm coated tip was irradiated with 365 or 654 nm lasers in a ∼ 95% RH environment, and the signal intensity was measured at the free end (Supplementary Movie 8). While the droplet size had minimal effect, an increase in droplet numbers reduced the light output significantly (Figure 6b, Supplementary Figure 20). A series of processes—coalescence, sliding, and droplet detachment—restored the original emission periodically (Figure 6c), reflecting the cyclic nature of the water collection process. This could serve as a method for tracking water collection frequency and assessing harvesting efficiency. In addition, a free-standing Janus crystal (TTP/P^2^/**1**)|**1**, due to its elasticity, bent up to 88° as droplets grew, and then straightened as the droplets fell off (Supplementary Figures 21, 22). In this dynamic process, the gravitational force pulling the water droplet down is balanced by the resilience of the crystal to flexural deformation. The bending and unbending occurred periodically as the humidity condensed, droplets grew, and the liquid was released (Supplementary Movie 9). Three-dimensional light intensity maps monitored the process, revealing three output signals corresponding to light decoupling at the contact interface with the two droplets and the crystal tip (Supplementary Figures 23–25). The signals shifted with droplet movement and condensation. These dynamic, self-sensing, bending crystals combine water collection and optical monitoring, enabling real-time tracking of the fog harvesting.

We also found that, by introducing two new phase boundaries, a water droplet on the crystal surface can be used to recover some of the light leaking from damaged crystals or to couple the light refracted from two bent crystals that act as optical transducers by using the evanescent wave.^53,54^ A crystal of TTP/P^2^/**3** was partially cleaved in the middle without complete separation, one of its ends was excited with light, and the signal was collected at the other end (Supplementary Figure 26a, b). The light dissipation at the crack decreased the output intensity to *I*/*I*_0_ = 29%. Covering the crack with a droplet of water recovered the light intensity of up to *I*/*I*_0_ = 37% due to partial light confinement within the droplet (Supplementary Figure 26c). To quantify the effect, a 2.3 cm-long crystal of TTP/P^2^/**3** with an optical loss coefficient α = 0.057 dB mm^–1^ (Supplementary Figures 27 and 28) was partially fractured about 2 mm from the output terminus, resulting in decreased intensity and α = 0.212 dB mm^–1^. Adding a droplet of water over the fracture improved the light transmission and reduced the optical loss to α = 0.132 dB mm^–1^, supporting the partially restorative role of water droplets on the optical performance of physically damaged crystals. Water droplets can also act as 2 × 2 couplers of four terminals from two crystals (Figure 6d). Two crystals, TTP/P^2^/**2**, each with a length of 4–5 mm, were fixed and bent into arcs with proximate convex sides and a gap of about 60 μm (Supplementary Figure 29), which prevented the coupling of the green fluorescence (493 nm). While covering the gap with a droplet of water guided 97.3% of the light intensity to the other end of the same crystal, a small portion of the light was transmitted to both terminals of the other crystal (Figure 6e, f). Excitation of the second crystal produced a similar result, with 96.3% of the light collected at its other end and the remaining light split between the two ends of the first crystal (Figure 6g–i). Although the coupling ratio is only about 1−2%, these results demonstrate that water could redirect or partially recover the emission of crystals that could be used as optical sensing elements.

## Conclusions

In summary, this study demonstrates the combined humidity-harvesting, signal-transmissive, and fog-collecting capabilities of organic crystals that were partially modified with multiple polyelectrolytes. The fog collection and retention can be detected optically by light decoupling at the solid–liquid interface, enabling real-time signal monitoring. The flexibility of the hybrid crystals attains active fog collection by bending to deliver liquid water. This innovative approach not only capitalizes on the mechanical compliance of organic crystals but also paves the way for the design of active, two-dimensional, self-sensing, and efficient harvesters, where the material can collect water and simultaneously report on the dynamics of the process, the latter being central to process optimization.

## Supporting Information Available

Experimental procedures, supplementary content, and figures (PDF)
Full legends for Supplementary Movies (PDF)
Supplementary Movie 1 showing comparison of the droplet mobility on the surface of a crystal of **1** at a 36° tilt and on the hybrid crystal TTP/P^2^/**1** at a 41° tilt. (MP4)
Supplementary Movie 2 showing fog capture and evaporation processes of a crystal of **2**. (MP4)
Supplementary Movie 3 showing fog capture and evaporation processes of a hybrid crystal of TTP/P^2^/**2**. (MP4)
Supplementary Movie 4 showing comparison of fog collection by a crystals of **1** and TTP/P^2^/**1**. (MP4)
Supplementary Movie 5 showing fog collection of Janus crystals (TTP/P^2^/**1**–**3**)**|1**–**3**. (MP4)
Supplementary Movie 6 showing fog collection of a wide lamellar Janus crystal (TTP/P^2^/**2**)**|2**. (MP4)
Supplementary Movie 7 showing fog collection of 60 Janus crystals (TTP/P^2^/**1**)**|1**. (MP4)
Supplementary Movie 8 showing passive optical transduction through a crystal of (TTP/P^2^/**2**)**|2** during fog collection. (MP4)
Supplementary Movie 9 showing the dynamic cyclic process in which droplets are collected at the tip of the Janus crystal (TTP/P^2^/**2**)**|2**, the crystal bends, and the droplets fall off the crystal. (MP4)

## References

[ref1] Wang Q.; Yang F.; Guo Z. The intrigue of directional water collection interface: mechanisms and strategies. J. Mater. Chem. A 2021, 9 (40), 22729–22758. 10.1039/D1TA06182H.

[ref2] Liu Z.; Liu H.; Li W.; Song J. Optimization of bioinspired surfaces with enhanced water transportation capacity. Chem. Eng. J. 2022, 433, 134568 10.1016/j.cej.2022.134568.

[ref3] Gu G.; Gu G.; Wang J.; Yao X.; Ju J.; Cheng G.; Du Z. A water collection system with ultra-high harvest rate and ultra-low energy consumption by integrating triboelectric plasma. Nano Energy 2022, 96, 107081 10.1016/j.nanoen.2022.107081.

[ref4] Wang B.; Zhou X.; Guo Z.; Liu W. Recent advances in atmosphere water harvesting: design principle, materials, devices, and applications. Nano Today 2021, 40, 101283 10.1016/j.nantod.2021.101283.

[ref5] Lu H.; Shi W.; Guo Y.; Guan W.; Lei C.; Yu G. Materials engineering for atmospheric water harvesting: progress and perspectives. Adv. Mater. 2022, 34 (12), 2110079 10.1002/adma.202110079. 35122451

[ref6] Shi W.; Guan W.; Lei C.; Yu G. Sorbents for atmospheric water harvesting: from design principles to applications. Angew. Chem. Int. Ed. 2022, 61, e202211267 10.1002/anie.202211267. 35960199

[ref7] Gao W.; Emaminejad S.; Nyein H. Y. Y.; Challa S.; Chen K.; Peck A.; Fahad H. M.; Ota H.; Shiraki H.; Kiriya D.; Lien D.-H.; Brooks G. A.; Davis R. W.; Javey A. Fully integrated wearable sensor arrays for multiplexed in situ perspiration analysis. Nature 2016, 529, 509–514. 10.1038/nature16521. 26819044 PMC4996079

[ref8] Thompson A. J.; Orué A. I. C.; Nair A. J.; Price J. R.; McMurtrie J.; Clegg J. K. Elastically flexible molecular crystals. Chem. Soc. Rev. 2021, 50 (21), 11725–11740. 10.1039/D1CS00469G. 34528036

[ref9] Wu W.; Chen K.; Wang T.; Wang N.; Huang X.; Zhou L.; Wang Z.; Hao H. Stimuli-responsive flexible organic crystals. J. Mater. Chem. C 2023, 11 (6), 2026–2052. 10.1039/D2TC04642C.

[ref10] Lan L.; Li L.; Naumov P.; Zhang H. Flexible organic crystals for dynamic optical transmission. Chem. Mater. 2023, 35 (18), 7363–7385. 10.1021/acs.chemmater.3c01659.

[ref11] Hayashi S.; Yamamoto S.; Takeuchi D.; Ie Y.; Takagi K. Creating elastic organic crystals of π-conjugated molecules with bending mechanofluorochromism and flexible optical waveguide. Angew. Chem. Int. Ed. 2018, 57 (52), 17002–17008. 10.1002/anie.201810422. 30341834

[ref12] Chandrasekar R. Mechanophotonics—mechanical micromanipulation of single-crystals toward organic photonic integrated circuits. Small 2021, 17 (24), 2100277 10.1002/smll.202100277. 33938127

[ref13] Lan L.; Liu H.; Tang B.; Yu X.; Liu X.; Zhang H. Polymer-coated organic crystals with solvent-resistant capacity and optical waveguiding function. Angew. Chem. Int. Ed. 2021, 60 (20), 11283–11287. 10.1002/anie.202102285. 33751744

[ref14] Kim D. W.; Hagiwara Y.; Hasebe S.; Dogan N. O.; Zhang M.; Asahi T.; Koshima H.; Sitti M. Broad-wavelength light-driven high-speed hybrid crystal actuators actuated inside tissue-like phantoms. Adv. Funct. Mater. 2023, 33 (47), 2305916 10.1002/adfm.202305916.

[ref15] Pantuso E.; Ahmed E.; Fontananova E.; Brunetti A.; Tahir I.; Karothu D. P.; Alnaji N. A.; Dushaq G.; Rasras M.; Naumov P.; Di Profio G. Smart dynamic hybrid membranes with self-cleaning capability. Nat. Commun. 2023, 14, 5751 10.1038/s41467-023-41446-9. 37717049 PMC10505219

[ref16] Lan L.; Li L.; Di Q.; Yang X.; Liu X.; Naumov P.; Zhang H. Organic single-crystal actuators and waveguides that operate at low temperatures. Adv. Mater. 2022, 34 (14), 2200471 10.1002/adma.202200471. 35104918

[ref17] Yang X.; Lan L.; Li L.; Yu J.; Liu X.; Tao Y.; Yang Q. H.; Naumov P.; Zhang H. Collective photothermal bending of flexible organic crystals modified with MXene-polymer multilayers as optical waveguide arrays. Nat. Commun. 2023, 14, 3627 10.1038/s41467-023-39162-5. 37336878 PMC10279756

[ref18] Yang X.; Lan L.; Li L.; Liu X.; Naumov P.; Zhang H. Remote and precise control over morphology and motion of organic crystals by using magnetic field. Nat. Commun. 2022, 13, 2322 10.1038/s41467-022-29959-1. 35484161 PMC9050695

[ref19] Peng J.; Han C.; Bai J.; Wang J.; Cao X.; Jia J.; Wang Y.; Wu J. Organic single crystal/polymer hybrid actuator with waveguide, low temperature, and humidity actuation properties. Cryst. Growth Des. 2022, 22 (12), 7187–7194. 10.1021/acs.cgd.2c00855.

[ref20] Lan L.; Yang X.; Tang B.; Yu X.; Liu X.; Li L.; Naumov P.; Zhang H. Hybrid elastic organic crystals that respond to aerial humidity. Angew. Chem. Int. Ed. 2022, 61 (14), e202200196 10.1002/anie.202200196. 35090063

[ref21] Yang X.; Lan L.; Pan X.; Di Q.; Liu X.; Li L.; Naumov P.; Zhang H. Bioinspired soft robots based on organic polymer-crystal hybrid materials with response to temperature and humidity. Nat. Commun. 2023, 14, 2287 10.1038/s41467-023-37964-1. 37085510 PMC10121608

[ref22] Yang X.; Lan L.; Pan X.; Liu X.; Song Y.; Yang X.; Dong Q.; Li L.; Naumov P.; Zhang H. Electrically conductive hybrid organic crystals as flexible optical waveguides. Nat. Commun. 2022, 13, 7874 10.1038/s41467-022-35432-w. 36550106 PMC9780324

[ref23] Lan L.; Li L.; Yang X.; Naumov P.; Zhang H. Repair and splicing of centimeter-size organic crystalline optical waveguides. Adv. Funct. Mater. 2023, 33 (11), 2211760 10.1002/adfm.202211760.

[ref24] Xu P.; Yu Q.; Chen Y.; Cheng P.; Zhang Z. Protective coating with crystalline shells to fabricate dual-stimuli responsive actuators. CCS Chem. 2022, 4 (1), 205–213. 10.31635/ccschem.021.202000663.

[ref25] Niazov-Elkan A.; Weissman H.; Dutta S.; Cohen S. R.; Iron M. A.; Pinkas I.; Bendikov T.; Rybtchinski B. Self-assembled hybrid materials based on organic nanocrystals and carbon nanotubes. Adv. Mater. 2018, 30 (2), 1705027 10.1002/adma.201705027. 29171679

[ref26] Eaby A. C.; Myburgh D. C.; Kosimov A.; Kwit M.; Esterhuysen C.; Janiak A. M.; Barbour L. J. Dehydration of a crystal hydrate at subglacial temperatures. Nature 2023, 616, 288–292. 10.1038/s41586-023-05749-7. 37045922 PMC10097597

[ref27] Saha S.; Mishra M. K.; Reddy C. M.; Desiraju G. R. From molecules to interactions to crystal engineering: mechanical properties of organic solids. Acc. Chem. Res. 2018, 51 (11), 2957–2967. 10.1021/acs.accounts.8b00425. 30351918

[ref28] Zhang T.; Zhou Z.; Liu X.; Wang K.; Fan Y.; Zhang C.; Yao J.; Yan Y.; Zhao Y. S. Thermally activated lasing in organic microcrystals toward laser displays. J. Am. Chem. Soc. 2021, 143 (48), 20249–20255. 10.1021/jacs.1c08824. 34797057

[ref29] Feng J.; Qiu Y.; Jiang L.; Wu Y. Long-range-ordered assembly of micro-/nanostructures at superwetting interfaces. Adv. Mater. 2022, 34 (9), 2106857 10.1002/adma.202106857. 34908188

[ref30] Yu L.; Chen G. Y.; Xu H.; Liu X. Substrate-independent, transparent oil-repellent coatings with self-healing and persistent easy-sliding oil repellency. ACS Nano 2016, 10 (1), 1076–1085. 10.1021/acsnano.5b06404. 26728655

[ref31] Liu P.; Zhang H.; He W.; Li H.; Jiang J.; Liu M.; Sun H.; He M.; Cui J.; Jiang L.; Yao X. Development of “liquid-like” copolymer nanocoatings for reactive oil-repellent surface. ACS Nano 2017, 11 (2), 2248–2256. 10.1021/acsnano.7b00046. 28192661

[ref32] Lyu P.; Zhang X.; Jiang X.; Shang B.; Liu X.; Deng Z. One-step preparation of hydrophobic surfaces containing hydrophilic groups for efficient water harvesting. Langmuir 2021, 37 (31), 9630–9636. 10.1021/acs.langmuir.1c01756. 34333978

[ref33] Jiang S.; Chen Q.; Tripathy M.; Luijten E.; Schweizer K. S.; Granick S. Janus particle synthesis and assembly. Adv. Mater. 2010, 22 (10), 1060–1071. 10.1002/adma.200904094. 20401930

[ref34] Li X.; Chen L.; Cui D.; Jiang W.; Han L.; Niu N. Preparation and application of Janus nanoparticles: recent development and prospects. Coord. Chem. Rev. 2022, 454, 214318 10.1016/j.ccr.2021.214318.

[ref35] Yang C.; Han N.; Han C.; Wang M.; Zhang W.; Wang W.; Zhang Z.; Li W.; Zhang X. Design of a Janus F-TiO_2_@PPS porous membrane with asymmetric wettability for switchable oil/water separation. ACS Appl. Mater. Interfaces 2019, 11 (25), 22408–22418. 10.1021/acsami.9b05191. 31149793

[ref36] Song Y.-y.; Liu Y.; Jiang H.-b.; Li S.-y.; Kaya C.; Stegmaier T.; Han Z.-w.; Ren L.-q. A bioinspired structured graphene surface with tunable wetting and high wearable properties for efficient fog collection. Nanoscale 2018, 10 (34), 16127–16137. 10.1039/C8NR04109A. 30117515

[ref37] Zhu H.; Guo Z. Hybrid engineered materials with high water-collecting efficiency inspired by Namib Desert beetles. Chem. Commun. 2016, 52 (41), 6809–6812. 10.1039/C6CC01894G. 27125658

[ref38] Hu Y.; Zhou M.; Fu H. Durable superhydrophobic coating based on halloysite nanotubes for versatile oil/water separation and reusable water collection. Surf. Interfaces 2024, 46, 104168 10.1016/j.surfin.2024.104168.

[ref39] Ji Y.; Yang W.; Li X.; Hou K.; Du P.; Zhao H.; Fan Z.; Xu B.; Cai Z. Thermodynamically induced interfacial condensation for efficient fog harvesting. Small 2023, 19 (46), 2304037 10.1002/smll.202304037. 37469016

[ref40] Wu J.; Yan Z.; Yan Y.; Li C.; Dai J. Beetle-inspired dual-directional Janus pumps with interfacial asymmetric wettability for enhancing fog harvesting. ACS Appl. Mater. Interfaces 2022, 14 (43), 49338–49351. 10.1021/acsami.2c14808. 36268797

[ref41] Wang X.; Zeng J.; Yu X.; Zhang Y. Superamphiphobic coatings with polymer-wrapped particles: enhancing water harvesting. J. Mater. Chem. A 2019, 7 (10), 5426–5433. 10.1039/C8TA12372A.

[ref42] Wang Y.; Zhang L.; Wu J.; Hedhili M. N.; Wang P. A facile strategy for the fabrication of a bioinspired hydrophilic-superhydrophobic patterned surface for highly efficient fog-harvesting. J. Mater. Chem. A 2015, 3 (37), 18963–18969. 10.1039/C5TA04930J.

[ref43] Wen C.; Guo H.; Bai H.; Xu T.; Liu M.; Yang J.; Zhu Y.; Zhao W.; Zhang J.; Cao M.; Zhang L. Beetle-inspired hierarchical antibacterial interface for reliable fog harvesting. ACS Appl. Mater. Interfaces 2019, 11 (37), 34330–34337. 10.1021/acsami.9b11862. 31429271

[ref44] Park H.; Hwang J.; Lee T. H.; Lee J.; Kang D. J. Fog collection based on secondary electrohydrodynamic-induced hybrid structures with anisotropic hydrophilicity. ACS Appl. Mater. Interfaces 2021, 13 (23), 27575–27585. 10.1021/acsami.1c04761. 34085809

[ref45] Bai H.; Wang L.; Ju J.; Sun R.; Zheng Y.; Jiang L. Efficient water collection on integrative bioinspired surfaces with star-shaped wettability patterns. Adv. Mater. 2014, 26 (29), 5025–5030. 10.1002/adma.201400262. 24847736

[ref46] Wang Q.; Yang F.; Guo Z. Design and construction of a Laplace and wettability gradient field for efficient water collection. Chem. Commun. 2023, 59 (40), 6048–6051. 10.1039/D3CC01104F. 37102965

[ref47] Wang Y.; Zhou Y.; Han P.; Qi G.; Gao D.; Zhang L.; Wang C.; Che J.; Wang Y.; Tao S. Improved water collection from short-term fog on a patterned surface with interconnected microchannels. Environ. Sci. Technol. 2024, 58 (8), 3812–3822. 10.1021/acs.est.3c09504. 38358300

[ref48] Zhu R.; Liu M.; Hou Y.; Zhang L.; Li M.; Wang D.; Wang D.; Fu S. Biomimetic fabrication of Janus fabric with asymmetric wettability for water purification and hydrophobic/hydrophilic patterned surfaces for fog harvesting. ACS Appl. Mater. Interfaces 2020, 12 (44), 50113–50125. 10.1021/acsami.0c12646. 33085450

[ref49] Moazzam P.; Tavassoli H.; Razmjou A.; Warkiani M. E.; Asadnia M. Mist harvesting using bioinspired polydopamine coating and microfabrication technology. Desalination 2018, 429, 111–118. 10.1016/j.desal.2017.12.023.

[ref50] Zhang Y.; Ming P.; Xue B.; Liu H.; Yang X.; Li L.; Niu S.; Yan L.; Zheng X.; Qin G. Facilely fabricating large-area robust heterogeneous wettability surface by mask-patterned ultrafine anode scanning electrodeposition for efficient water collection. Surf. Interfaces 2023, 41, 103247 10.1016/j.surfin.2023.103247.

[ref51] Liu H.; Lu Z.; Zhang Z.; Wang Y.; Zhang H. Highly elastic organic crystals for flexible optical waveguides. Angew. Chem. Int. Ed. 2018, 57 (28), 8448–8452. 10.1002/anie.201802020. 29752768

[ref52] Asano Y.; Zheng Y.; Nishino K.; Sato I. Depth sensing by near-infrared light absorption in water. IEEE Trans. Pattern Anal. Mach. Intell. 2020, 43 (8), 2611–2622. 10.1109/TPAMI.2020.2973986. 32078532

[ref53] Taitt C. R.; Anderson G. P.; Ligler F. S. Evanescent wave fluorescence biosensors: advances of the last decade. Biosens. Bioelectron. 2016, 76, 103–112. 10.1016/j.bios.2015.07.040. 26232145 PMC5012222

[ref54] Annadhasan M.; Agrawal A. R.; Bhunia S.; Pradeep V. V.; Zade S. S.; Reddy C. M.; Chandrasekar R. Mechanophotonics: flexible single-crystal organic waveguides and circuits. Angew. Chem. Int. Ed. 2020, 59 (33), 13852–13858. 10.1002/anie.202003820. 32392396

