# Supplementary material for: Efficient Aerial Water Harvesting with Self-Sensing Dynamic Janus Crystals
Source: J Am Chem Soc. 2024 Oct 22;146(44):30529–38. doi: 10.1021/jacs.4c11689 (PMC11544689; doi:10.1021/jacs.4c11689)
Supplement: Supplementary file 10 — ja4c11689_si_010.pdf [file ja4c11689_si_010.pdf]

Legends for the supplementary movies

Supplementary Movie 1. Comparison of the droplet fluidity on the surface of original crystal **1** at 36° tilt and the hybrid crystal TTP/P^2/**1** at 41° tilt.

Supplementary Movie 2. The water capture and evaporation processes of a crystal **2**. A conventional ultrasonic humidifier with fog-generating power of ~0.25 L h^{-1} was used to evaluate the fog-harvesting performance. The distance between the sample and the outlet was 5 cm. The temperature and relative humidity (RH) were 20 °C and ~95%, respectively.

Supplementary Movie 3. The water capture and evaporation processes of a hybrid crystal TTP/P^2/**2**. A conventional ultrasonic humidifier with fog-generating power of ~0.25 L h^{-1} was used to evaluate the fog-harvesting performance. The distance between the sample and the outlet was 5 cm. The temperature and relative humidity (RH) were 20 °C and ~95%, respectively.

Supplementary Movie 4. Comparison of water collection processes of the original crystal **1** and TTP/P^2/**1**. To more clearly reflect the difference in water collection rate, the distance between the fog flow outlet and the crystal was ~1.2 cm.

Supplementary Movie 5. Water collection with Janus crystals (TTP/P^2/**1**–**3**)|**1**–**3**. To more clearly reflect the difference in water collection rate, the distance between the fog flow outlet and the crystal was ~1.2 cm.

Supplementary Movie 6. The water collection processes of a wide lamellar Janus crystal (TTP/P^2/**2**)|**2**. To more clearly reflect the difference in water collection rate, the distance between the fog flow outlet and the crystal was ~1.2 cm.

Supplementary Movie 7. The water collection processes of 60 Janus crystal (TTP/P^2/**1**)|**1**. A conventional ultrasonic humidifier with fog-generating power of ~0.18 L h^{-1} was used to evaluate the fog-harvesting performance. The distance between the sample and the outlet was 9 – 10 cm. The temperature and relative humidity (RH) were 20 °C and ~85%, respectively.

Supplementary Movie 8. Passive optical transduction through a (TTP/P^2/**2**)|**2** during water collection. A conventional ultrasonic humidifier with fog-generating power of ~0.25 L h^{-1} was used to assess the humidity-harvesting performance. The distance between the samples and the outlet was 5 cm.

Supplementary Movie 9. The dynamic cyclic process in which droplets are collected at the tip of the Janus crystal (TTP/P^2/**2**)|**2**, the crystal bends, and the droplets fall off the crystal.
