# Supplementary material for: Efficient Aerial Water Harvesting with Self-Sensing Dynamic Janus Crystals
Source: J Am Chem Soc. 2024 Oct 22;146(44):30529–38. doi: 10.1021/jacs.4c11689 (PMC11544689; doi:10.1021/jacs.4c11689)
Supplement: Supplementary file 11 — ja4c11689_si_011.pdf [file ja4c11689_si_011.pdf]

Supporting Information

Efficient Aerial Water Harvesting with Self-Sensing Dynamic Janus Crystals

Linfeng Lan,[a,b] Liang Li,[c,d] Chenguang Wang,[b] Panče Naumov,[c,e,f,g,]* and Hongyu Zhang[a,]*

[a]*State Key Laboratory of Supramolecular Structure and Materials, College of Chemistry, Jilin University, Changchun, 130012, P. R. China*

[b]*State Key Laboratory of Integrated Optoelectronics, College of Electronic Science and Engineering, Jilin University, Changchun, 130012, P. R. China*

[c]*Smart Materials Lab, New York University Abu Dhabi, PO Box 129188, Abu Dhabi, UAE*

[d]*Department of Sciences and Engineering Department, Sorbonne University Abu Dhabi, PO Box 38044, Abu Dhabi, UAE*

[e]*Center for Smart Engineering Materials, New York University Abu Dhabi, PO Box 129188, Abu Dhabi, UAE*

[f]*Research Center for Environment and Materials, Macedonian Academy of Sciences and Arts, Bul. Krste Misirkov 2, MK–1000 Skopje, Macedonia*

[g]*Molecular Design Institute, Department of Chemistry, New York University, 100 Washington Square East, New York, NY 10003, USA*

*Corresponding authors. Emails for correspondence: pance.naumov@nyu.edu; hongyuzhang@jlu.edu.cn

The PDF file includes:

Materials and Methods
Supplementary Figures 1 to 29
Supplementary Tables 1 to 9
References

Other Supplementary Materials for this manuscript include the following:

Supplementary Movies 1 to 9

Table of Contents

1. Supplementary methods **3**

2. Supplementary figures **6**

3. Supplementary tables **20**

4. Legends for the supplementary movies **29**

5. Supplementary references **30**

1. Supplementary methods

Materials preparation. All materials for the syntheses were obtained from Energy Chemical and used as received. (*Z*)-3-(furan-2-yl)-2-(4-(((*E*)-2-hydroxy-5-methylbenzylidene)-amino)phenyl)acrylonitrile (compound **1**; MW: 328.37) and (*Z*)-4-(2-cyano-2-(4-(trifluoromethoxy)phenyl)vinyl)benzonitrile (compound **3**; MW: 314.27) were synthesized according to reported procedures.[1,2] 2,2'-((1*E*,1'*E*)-1,4-phenylenebis(ethene-2,1-diyl))-dibenzonitrile (compound **2**; MW: 332.14) was purchased from Energy Chemical and purified by column chromatography. Poly(diallyldimethylammonium chloride) solution (PDDA; average MW: 200000-350000; 20 wt. % in H_2O) and poly(styrene sulfonic acid) sodium (PSS; average MW: 70000) were purchased from Sigma-Aldrich and Alfa Aesar and used as received without purification. Tetraethyl orthosilicate (TEOS; MW: 208.33), triethoxy(3-glycidyloxypropyl)silane (TGOS; MW: 278.42) and bis(3-aminopropyl)-terminated poly(dimethylsiloxane) (H_2N–PDMS–NH_2; average MW: 3000) were purchased from Energy Chemical and used as received without purification.

Crystal growth. To prepare crystals of **1**, a series of saturated solutions of the compound in dichloromethane (DCM; 10 mmol L^{-1}) were added to test tubes, and an approximately identical volume of ethanol was carefully added along the tube wall. Needle-shaped crystals of **1** were obtained after allowing for a slow diffusion for a week at room temperature. Crystals of **2** and **3** were prepared by adding petroleum ether onto the top of dilute solutions of the respective compounds in DCM (~8 and ~5 mmol L^{-1}, respectively). The length of the as-crystallized samples of **1**–**3** ranged from several millimeters to several centimeters, and their thickness and width varied from tens to thousands of microns. The stacking of the molecules and the abundance of weak interactions are thought to contribute[3,4] to their mechanical compliance.

Hybridization of the organic crystals. The protocol for surface modification of the crystals is illustrated in Fig. S2. One of the ends of the crystals was vertically fixed on glass slides and alternatively dipped in solutions of PDDA and PSS, five times.[5] The samples were rinsed with distilled water after each soak and dried. Subsequently, they were immersed in a solution containing TEOS, TGOS, and H_2N–PDMS–NH_2 for twenty minutes.[6] The dipping solution was prepared by mixing 0.5 mmol H_2N–PDMS–NH_2, 5 mmol TEOS, and 5 mmol TGOS in a 25 mL round-bottom flask and stirring for 24 h. The crystals were then slowly lifted and transferred into a desiccator over hydrochloric acid (1 mL) for 9 hours to facilitate the hydrolysis and condensation of the polymers. The grafting was performed by an efficient reaction between the amine and epoxy groups and the hydrolysis and condensation of the ethoxy group in the presence of HCl vapors. During this process, TEOS and TGOS can be co-hydrolyzed to enhance the strength of the surface coating, while some of the free PDMS segments form a structure similar to a polymer brush.[6] The crystals were then thermally annealed at 60 °C for 12 h to obtain the hybrid crystals TTP/P^2/**1**–**3** and the Janus crystals (TTP/P^2/**1**–**3**)|**1**–**3**. For the latter, only half of the crystal was repeatedly and alternately soaked in aqueous solutions of PDDA and PSS. The coated sector was immersed in the silanizing solution (TGOS, TEOS, and H_2N–PDMS–NH_2).

Contact angle measurement. Most crystals of **1**–**3** were too small to measure the contact angle, Θ_{CA}. Instead, wide lamellar crystals **2** with a width >5 mm were utilized for measuring the static contact angles (Θ_{SCA}), sliding angles (Θ_{SA}), and contact angle hysteresis (Θ_{CAH}) using the DSA30 Drop Shape Analyzer (Krüss). The water droplets used for the measurement of Θ_{SCA} and Θ_{CAH} were 4 μL, and 20 μL for the measurement of Θ_{SA}. Θ_{SA} is defined as the minimum angle at which a droplet can slide on the crystal surface. At this angle, Θ_{CAH} is defined as the value of the advancing contact angle (Θ_{A}) minus the receding contact angle (Θ_{R}, Fig. S7).

Fog harvesting. A conventional ultrasonic humidifier with fog generation of ∼0.25 L h^{-1} was used to continuously provide a high-humidity environment for assessing the fog harvesting performance. All samples of original crystals and hybrid crystals were placed vertically, maintaining a distance of 5 cm between the samples and the outlet, to collect fog in an open system. The temperature and relative humidity (RH) were 20 °C and ~95%, respectively (environments with ~85% and ~75% RH were obtained by reducing the fog flow). Each cycle lasted 0.5 h, and the final water collection efficiency (r) was calculated by using Eq. 1:

$$r = (m - m_0)/(s \times t) \qquad \text{(Eq. 1)}$$

Where m_0 and m represent the weight of the beaker before and after fog collection, respectively. s represents the crystal area used to collect the mist, and t is the time of collection.

Characterization. Optical photographs of crystals are obtained by using either a Canon camera or an optical Olympus BX61 microscope. Emission spectra were recorded by a Maya2000 Pro CCD spectrometer. The three-point bending tests were carried out using an Instron 5944 universal testing system with a capacity of 5 N Instron 2530 load cells. Contact angles were measured using a KRUSS DSA 30 contact angle system. Scanning electron microscopic images were obtained on FEI Quanta 450 environmental scanning electron microscope (ESEM) and Regulus8100 field emission scanning electron microscope operating at 5 – 10 kV. Morphologies showing surface roughness were obtained on a Bruker Icon XR atomic force microscope working in tapping mode. The IR spectra were obtained on a Bruker 80V-ATR vacuum Fourier transform infrared spectrometer.

Optical measurements. Total internal reflection (TIR) of light occurs within a crystal under two conditions: (1) the refractive index of the crystal material must be higher than that of the surrounding medium; and (2) the incident angle of light entering the waveguide must be greater than the critical angle (θ_c).[12] According to the refraction law (Eqs. 2 and 3):

$$n_1 \times \sin\theta_1 = n_2 \times \sin\theta_2 \qquad \text{(Eq. 2)}$$
$$\theta_c = \sin^{-1}(n_2/n_1) \qquad \text{(Eq. 3)}$$

Where n_1 is the refractive index of the crystal (~1.6), n_2 is the refractive index of the medium, which can be air (1.0) or water (1.3), θ_1 is the incident angle, and θ_2 is the refracted angle. As

shown in Figure 5a, the critical angle is ~38.7° when air is the medium. Total internal reflection occurs within the crystal when the incident angle is greater than ~38.7°, while for angles less than ~38.7°, the light wave undergoes refraction and partially dissipates into the air. However, in the presence of water droplets on the crystal surface, the critical angle is ~56.2°. When the incident angle is less than ~56.2°, light undergoes refraction at the interface between the water droplet and the crystal, resulting in the output of the signal at the drop and increasing the optical loss.

For the optical waveguide tests, the crystal was irradiated by the third harmonic (355 nm) of a Nd:YAG (yttrium-aluminum-garnet) laser with a pulse duration of about 5 ns. The energy of the laser was adjusted by using calibrated neutral density filters. The beam was focused into a stripe whose shape was adjusted to 0.5 × 0.5 mm by using a plano-convex lens and a slit. The crystal was placed on a silicon wafer, and one tip of the crystal extended out of the edge of the wafer to align with the probe of the spectrometer. While changing the irradiate locations, spectral data were collected for each irradiated location at the excitation site and the tip of the crystal. All emission spectra were recorded on a Maya2000 Pro CCD spectrometer. Light intensity maps were obtained by mesh function analysis in MATLAB.

2. Supplementary figures

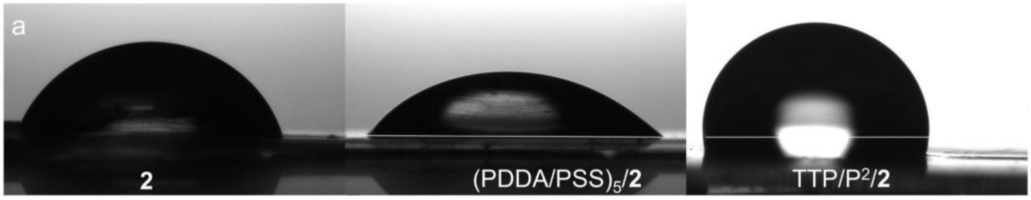

b

Sample	Θ_{SCA} / °					Average value
**2**	69.5	70.6	71.9	70.8	69.9	70.5 ± 0.9
(PDDA/PSS)$_5$/**2**	47.2	48.0	45.8	47.0	46.4	46.9 ± 0.8
TTP/P^2/**2**	96.8	95.5	97.4	96.3	95.8	96.4 ± 0.8

Supplementary Figure 1. Photographs (a) and water static contact angles (Θ_{SCA}, b) of crystals **2**, (PDDA/PSS)$_5$/**2** and TTP/P^2/**2**.

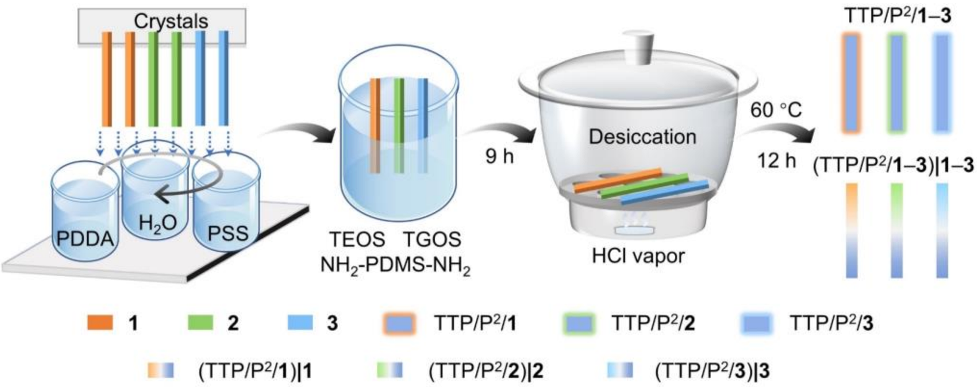

Supplementary Figure 2. Protocol for fabrication of the fog-collecting functional hybrid crystals. Complete immersion of the crystals results in the hybrid crystals TTP/P^2/**1**–**3**, while partial immersion affords Janus crystals (TTP/P^2/**1**–**3**)|**1**–**3**.

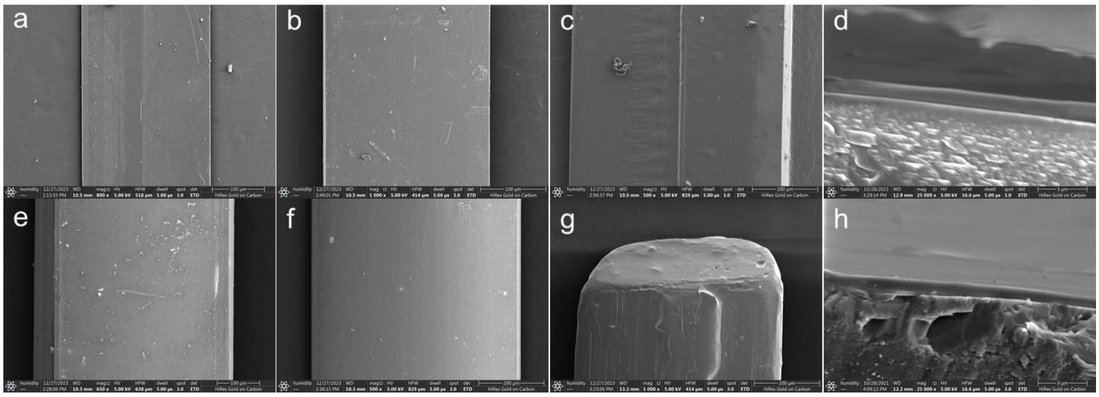

Supplementary Figure 3. Scanning electron micrographs showing surfaces of **1** (a) and **2** (e), (PDDA/PSS)$_5$/**1** (b) and (PDDA/PSS)$_5$/**2** (f), TTP/P^2/**1** (c) and TTP/P^2/**2** (g), and cross-sections of TTP/P^2/**1** (d) and TTP/P^2/**2** (h).

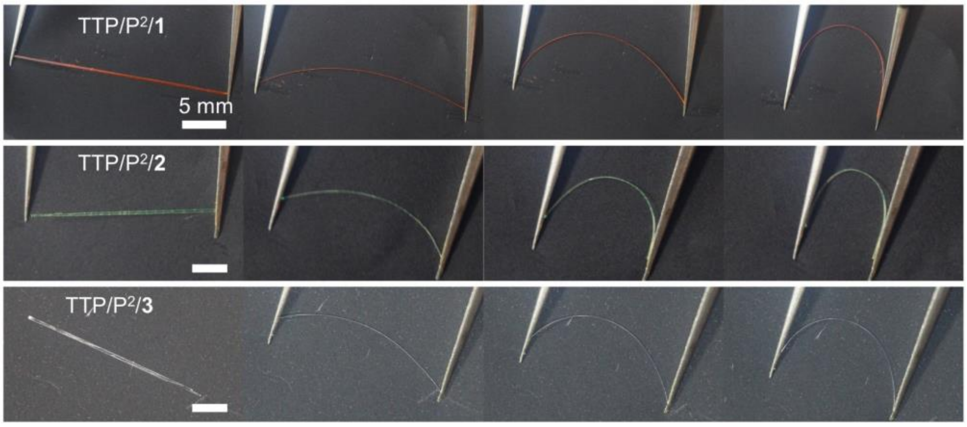

Supplementary Figure 4. A simple manual test of the mechanical elasticity of the hybrid crystals TTP/P^2/**1**–**3**. The crystals can be bent repeatedly when squeezed by a pair of tweezers.

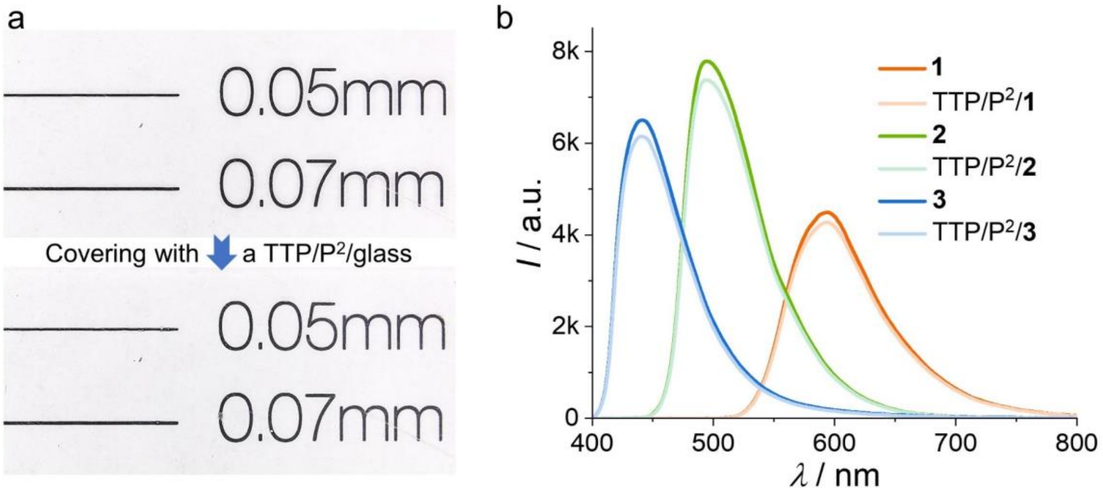

Supplementary Figure 5. (a) A graticule before and after covering with TTP/P^2 showing the optical transparency of the polymer film. (b) Fluorescence emission spectra of the original crystals **1**–**3** and the hybrid crystals TTP/P^2/**1**–**3** in the crystalline state under 365 nm excitation.

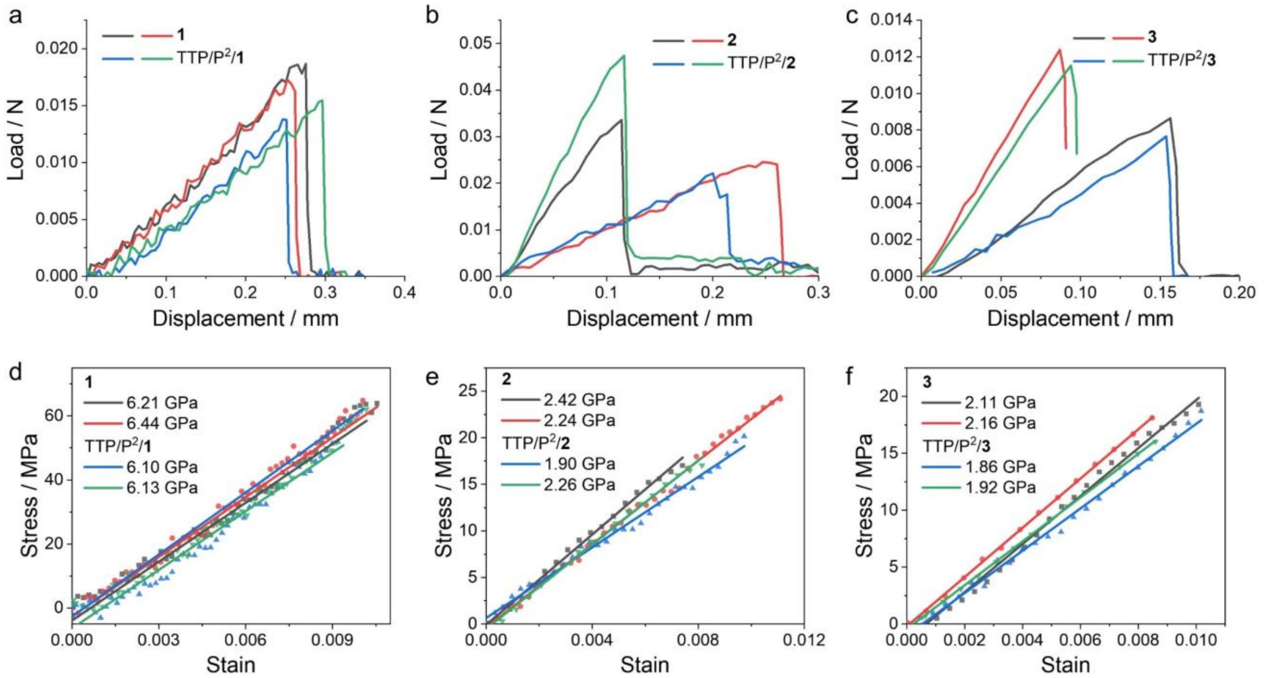

Supplementary Figure 6. (a–c) Displacement-load curves of crystals **1**–**3** and TTP/P^2/**1**–**3** obtained from three-point bending tests. (d–f) Corresponding stress-strain curves of crystals **1**–**3** and TTP/P^2/**1**–**3** obtained from three-point bending tests. The correlation coefficients are all greater than 0.97. All curves were derived from the same crystals of **1**–**3** which were cut into four pieces.

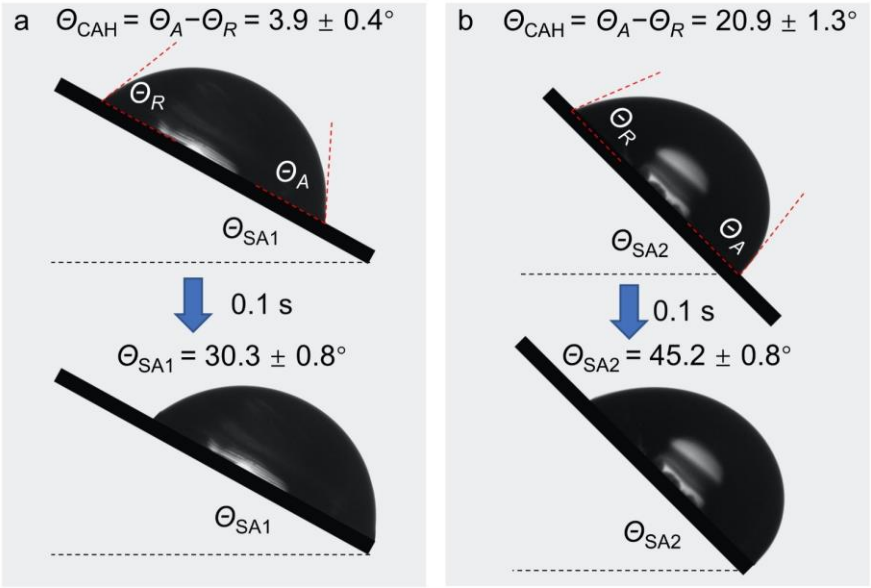

Supplementary Figure 7. Time-lapse snapshots of a sliding 20 μL water droplet on the wide lamellar crystal **2** (a) and TTP/P^2/**2** (b), showing the definition and measured values of the sliding angle (θ_{SA}) and contact angle hysteresis (θ_{CAH}).

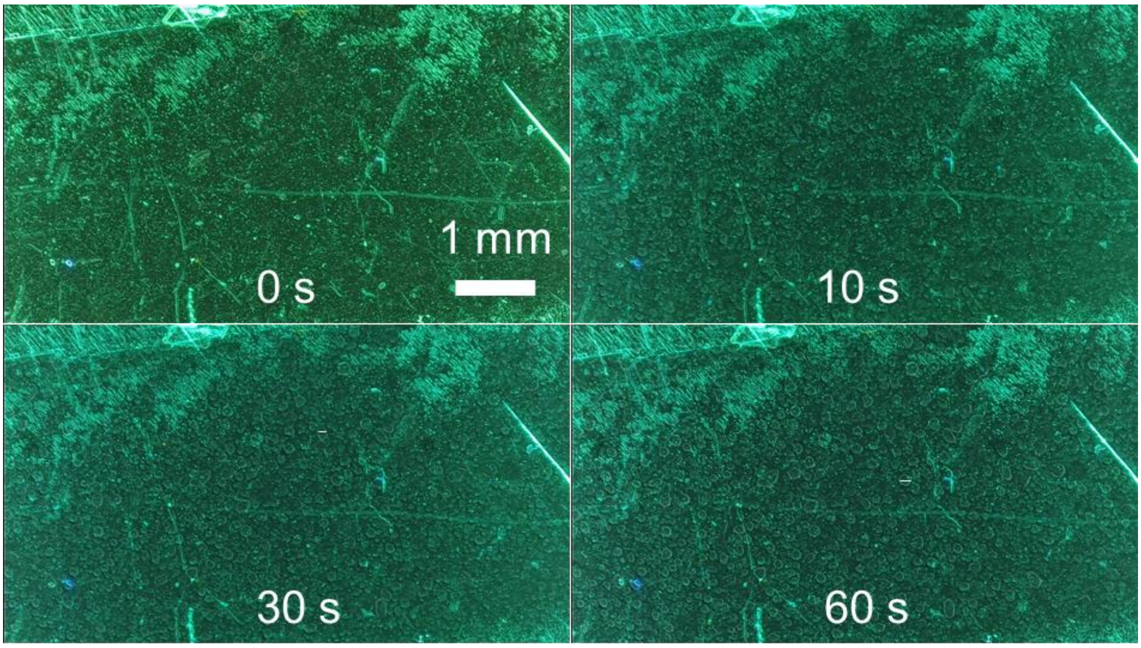

Supplementary Figure 8. Water droplets condensing on the surface of **2** at room temperature under UV light over a period of 60 s.

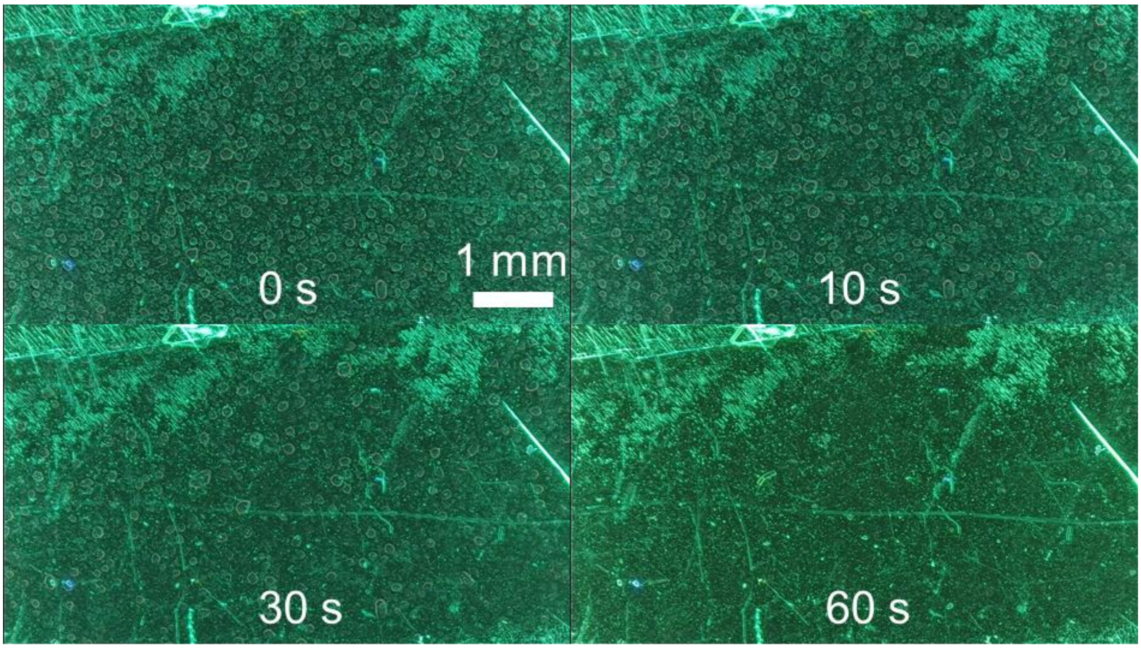

Supplementary Figure 9. Evaporation of water droplets on the surface of TTP/P^2/**2** at room temperature under UV light over a period of 60 s.

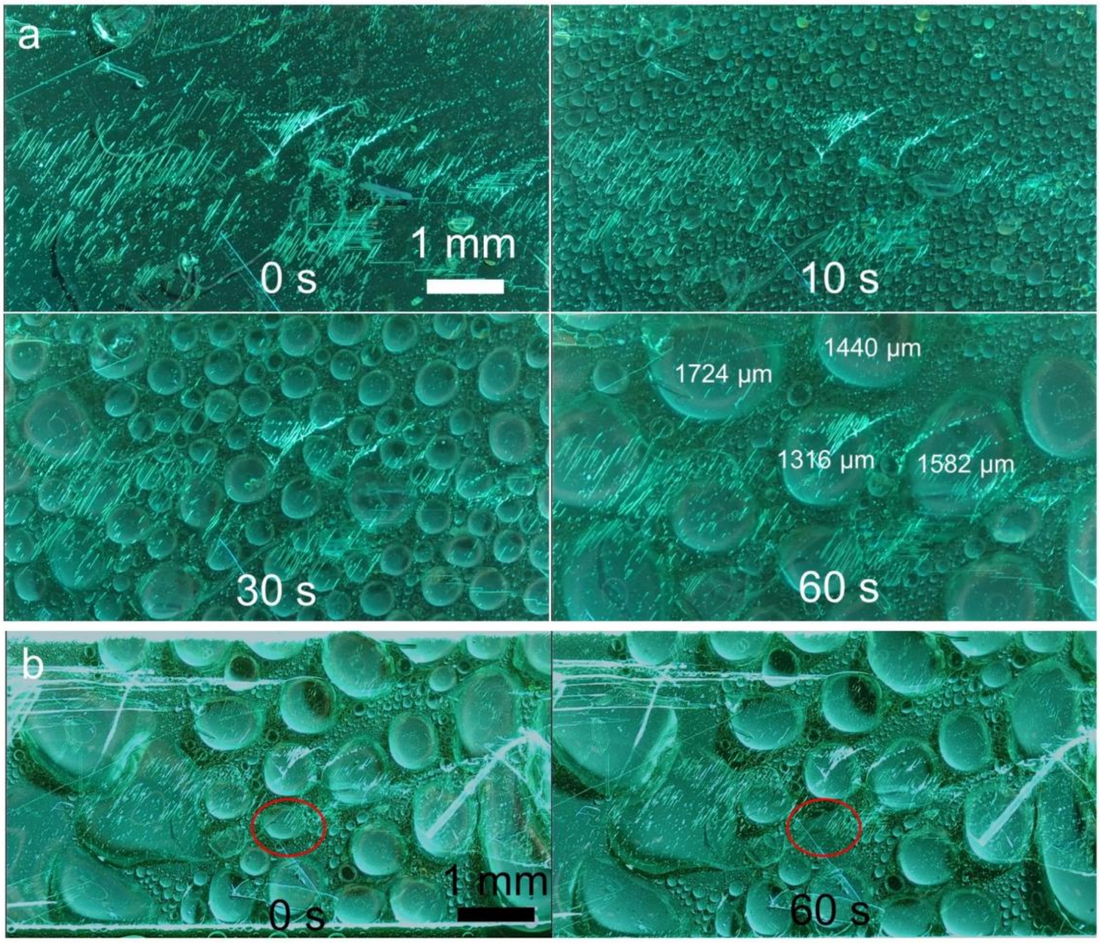

Supplementary Figure 10. Water droplets condensing (a) and evaporating (b) at the surface of TTP/P^2/**2** at room temperature under UV light over a period of 60 s.

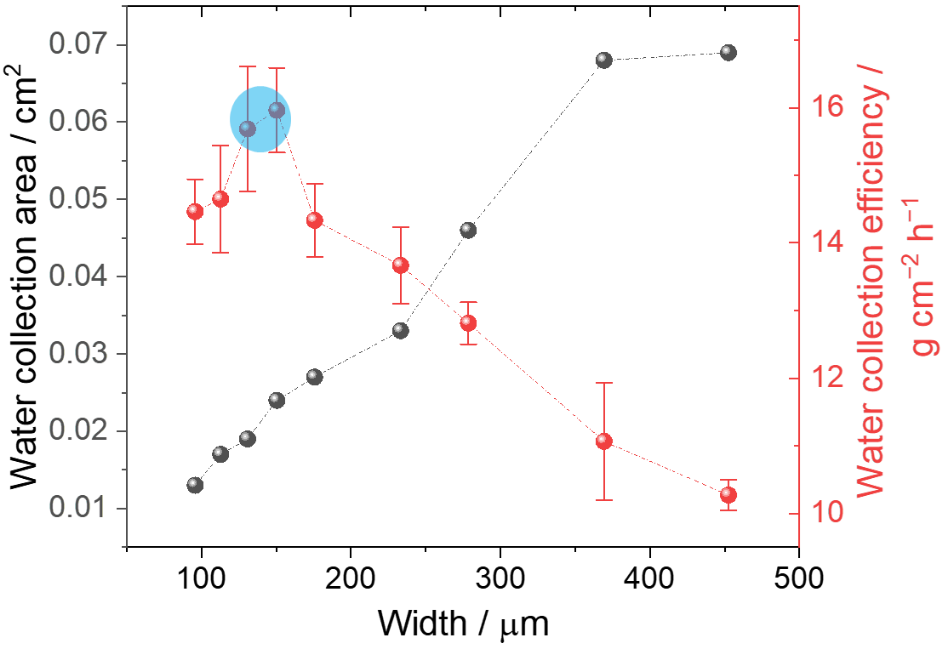

Supplementary Figure 11. The relationship between width, water collection area, and water collection efficiency of (TTP/P^2/**1**)|**1**. Each sample was tested three times.

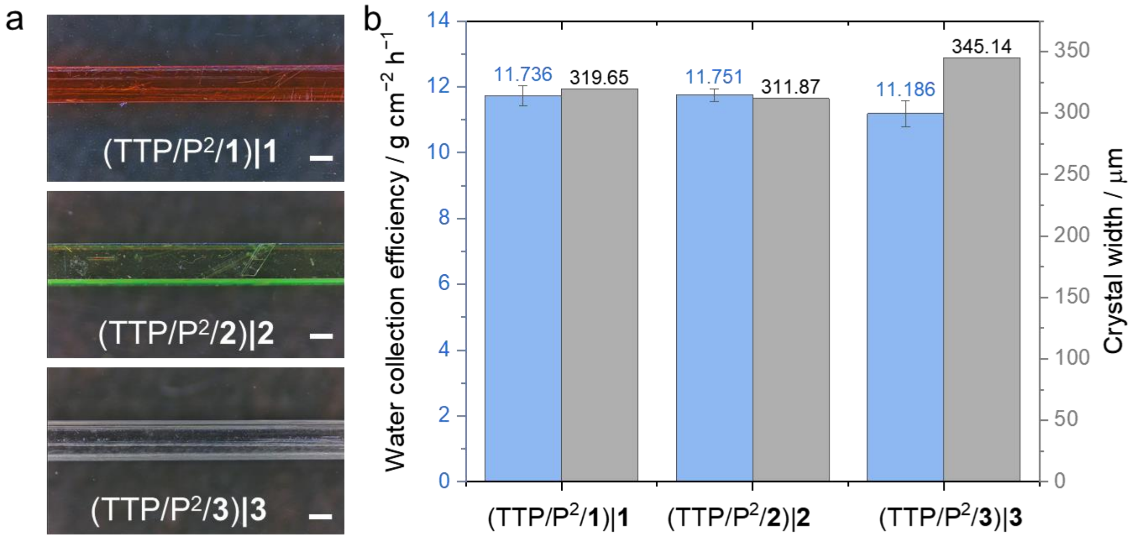

Supplementary Figure 12. (a) Microphotographs and (b) comparison of water collection efficiency of the Janus crystals (TTP/P^2/**1–3**)|**1–3** with similar width.

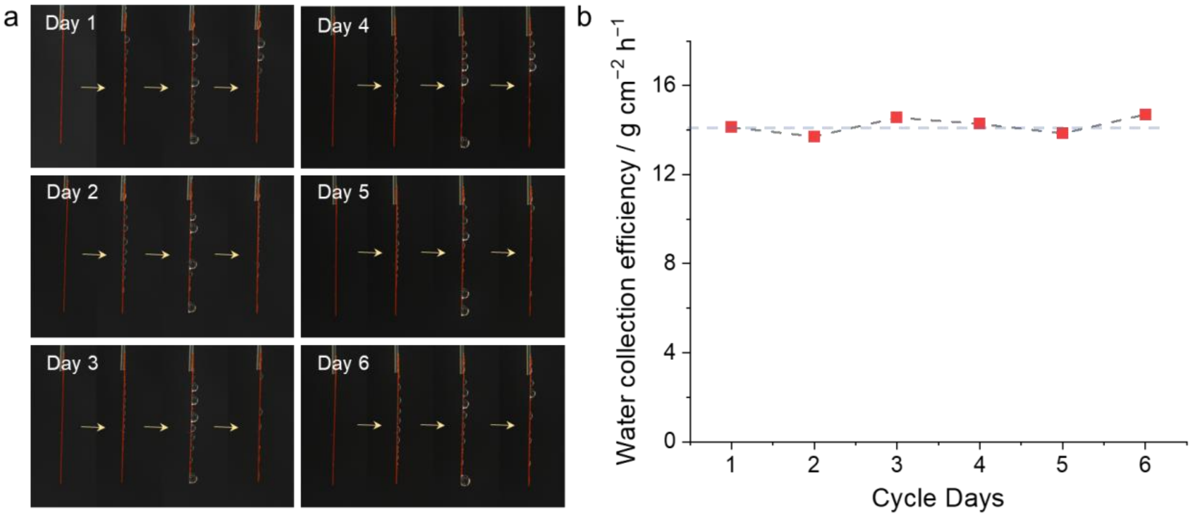

Supplementary Figure 13. (a) Photograph of the Janus crystal (TTP/P^2/**1**)|**1** showing the water collection process over six consecutive days. (b) Water collection efficiency changes of the (TTP/P^2/**1**)|**1** over six days.

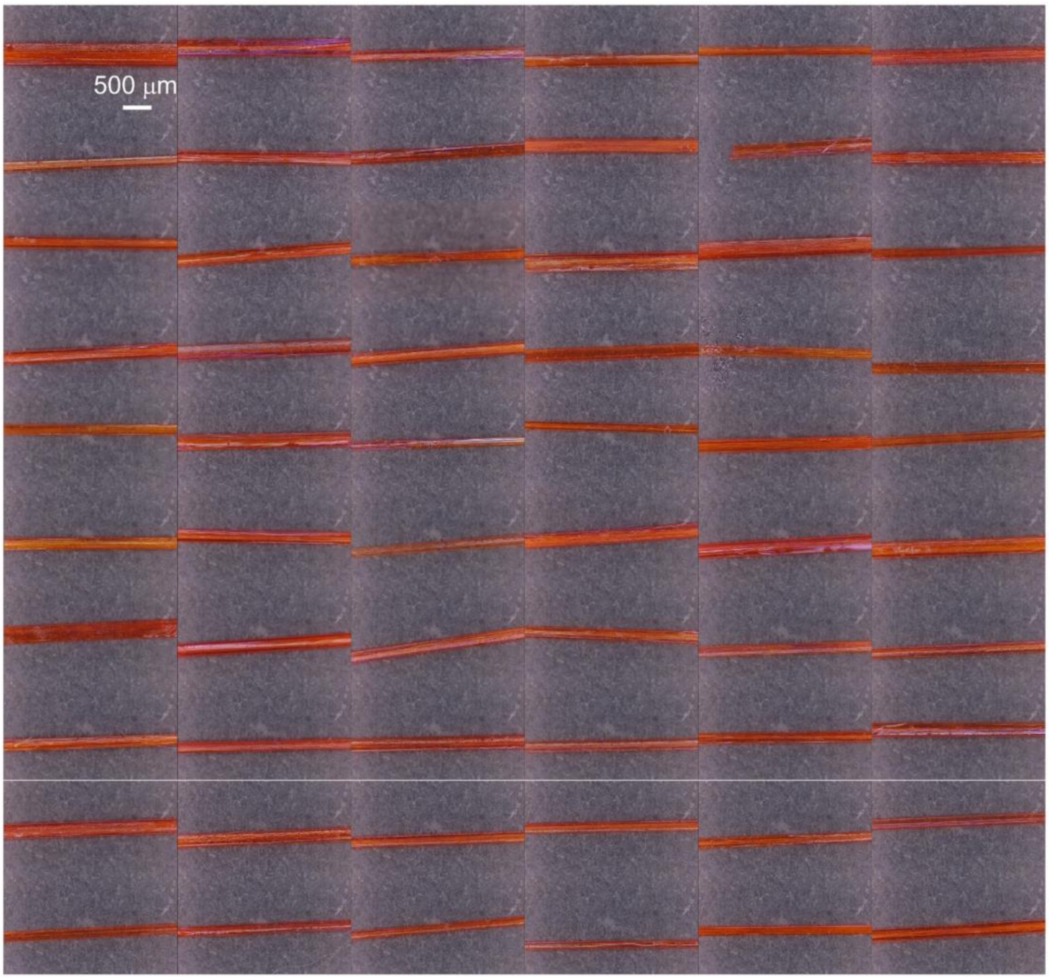

Supplementary Figure 14. Microscope photographs of 60 Janus crystals (TTP/P^2/**1**)|**1** showing their width.

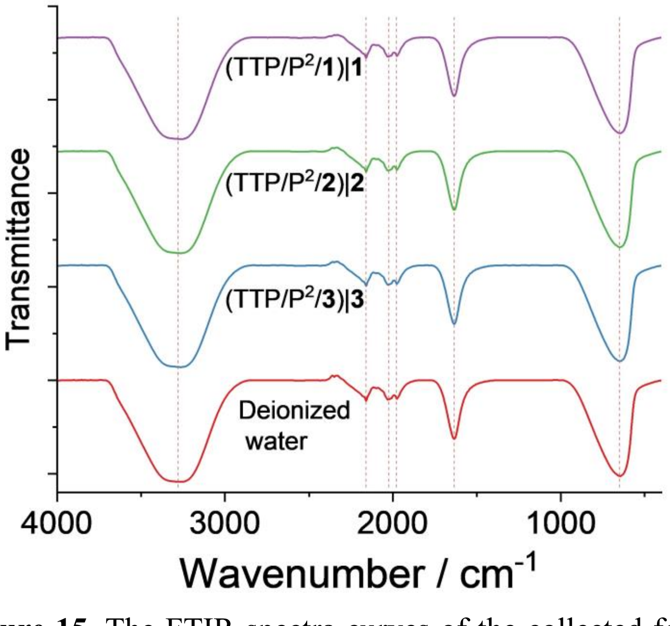

Supplementary Figure 15. The FTIR spectra curves of the collected fog water using Janus crystals (TTP/P^2/**1–3**)|**1–3** and deionized water.

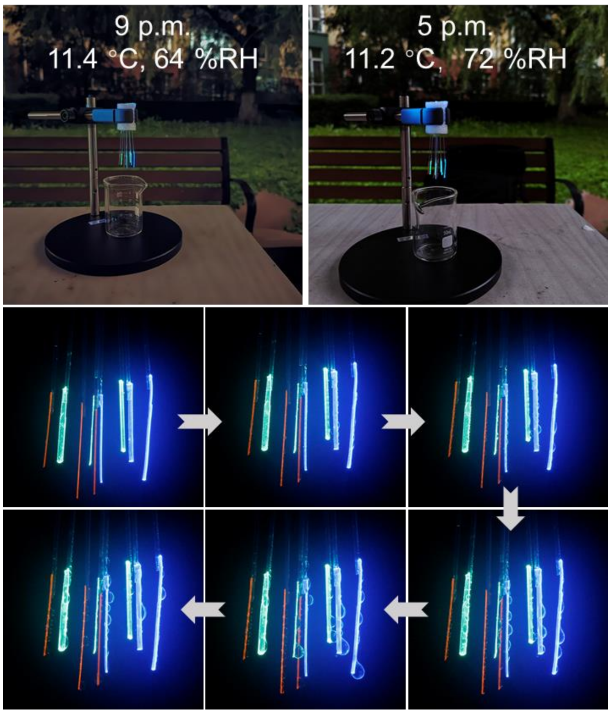

Supplementary Figure 16. Photographs of nine Janus crystals (TTP/P^2/**1–3**)|**1–3** placed outdoors to collect fog at night (from 9 p.m. to 5 a.m.) and the process of capturing water droplets at 3 a.m. Relative humidity ranges from 64 to 72 % throughout the night, with temperatures ranging from 9.5 °C to 11.4 °C and wind rating 1 to 3.

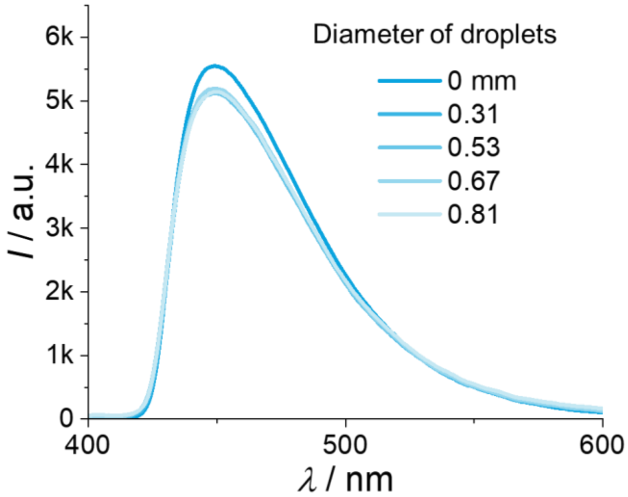

Supplementary Figure 17. The spectra collected at the end of a crystal of TTP/P^2/**3** when water droplets of different sizes are laid on the crystal surface.

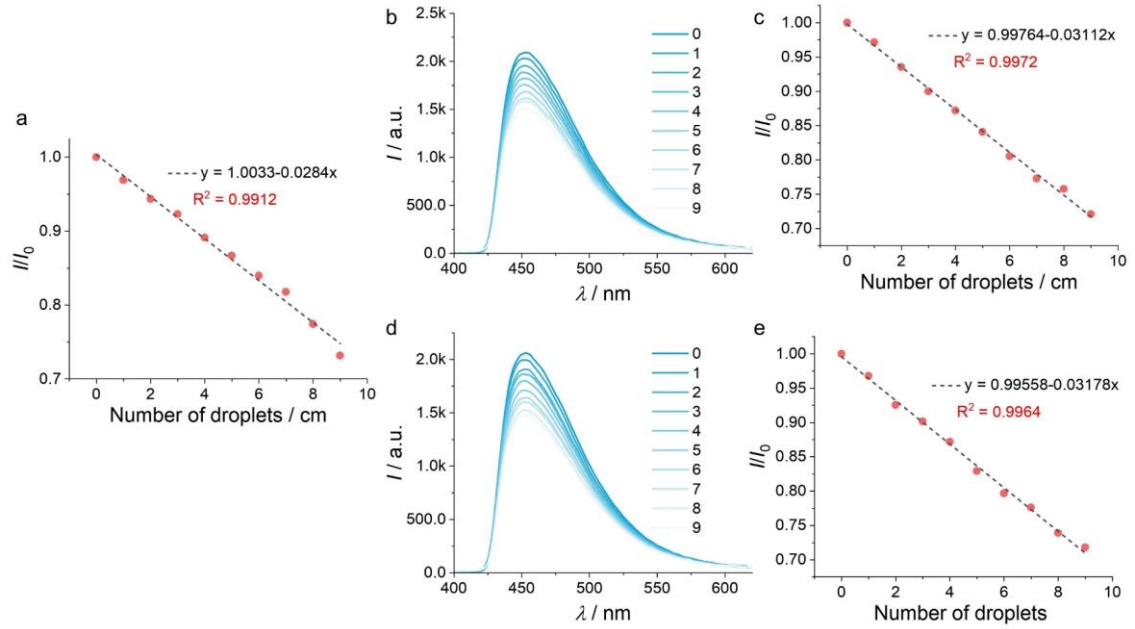

Supplementary Figure 18. Effect of the number of droplets on active optical waveguide of crystal. (a) The fitted linear curve of different numbers of droplets and signal intensity ratio (I/I_0) obtained from panel f of Figure 5. (b, c) The corresponding spectra (b) showing output signal changes, and the fitted linear curves (c) of different number of droplets and signal intensity ratio (I/I_0) obtained by the second repeated test of TTP/P^2/**3**. (d, e) The corresponding spectra (d) showing output signal changes, and the fitted linear curves (e) of different number of droplets and signal intensity ratio (I/I_0) obtained by the third repeated test of TTP/P^2/**3**.

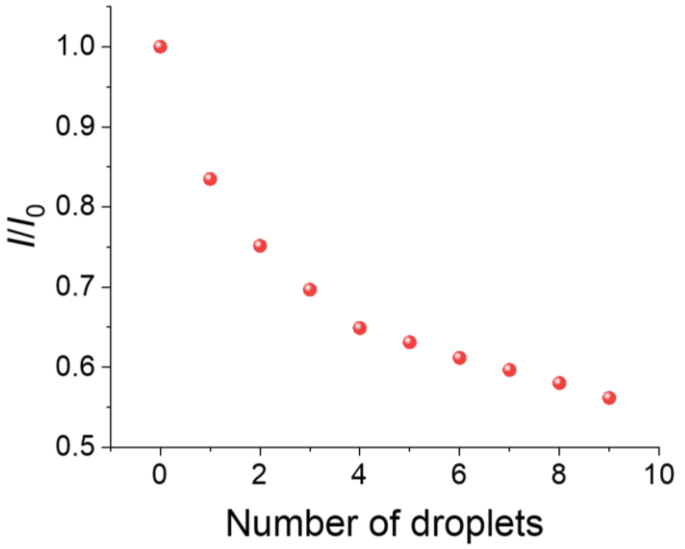

Supplementary Figure 19. Dependence of the signal intensity ratio (I/I_0) on the number of droplets by passive transduction of light through the crystal TTP/P^2/**3**. The crystal was excited under a 654 nm laser.

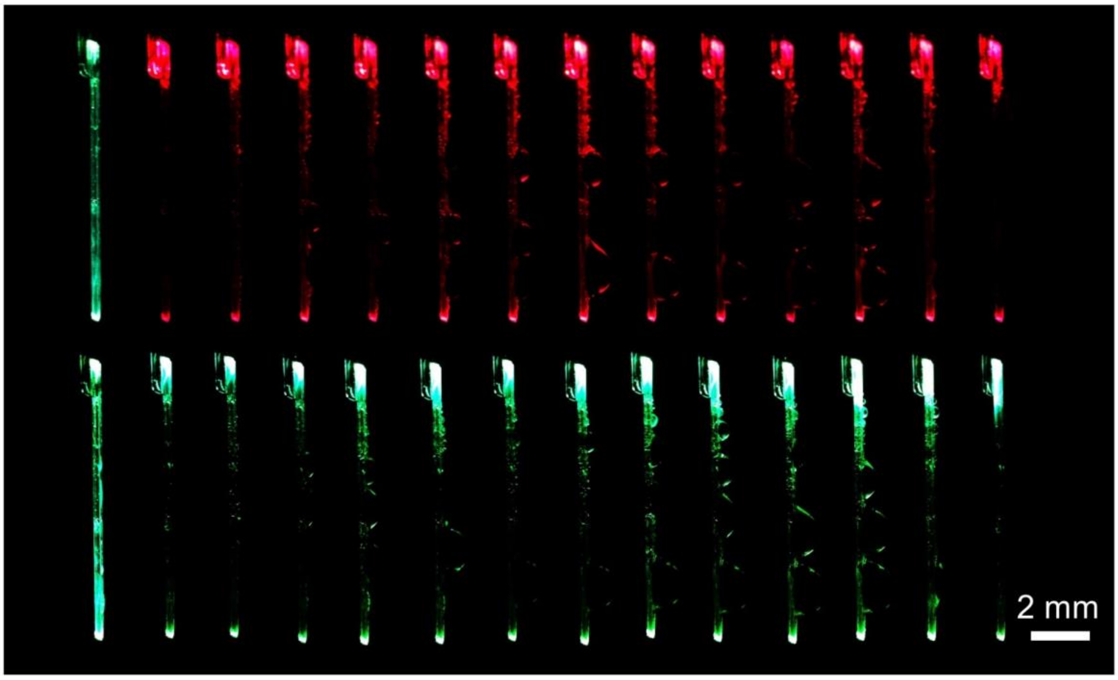

Supplementary Figure 20. Passive (top) and active (bottom) optical waveguiding by a Janus crystal (TTP/P^2/**2**)|**2** during fog collection over 20 s. The hybrid crystals were excited by 654 nm and 355 nm lasers for passive and active waveguiding, respectively.

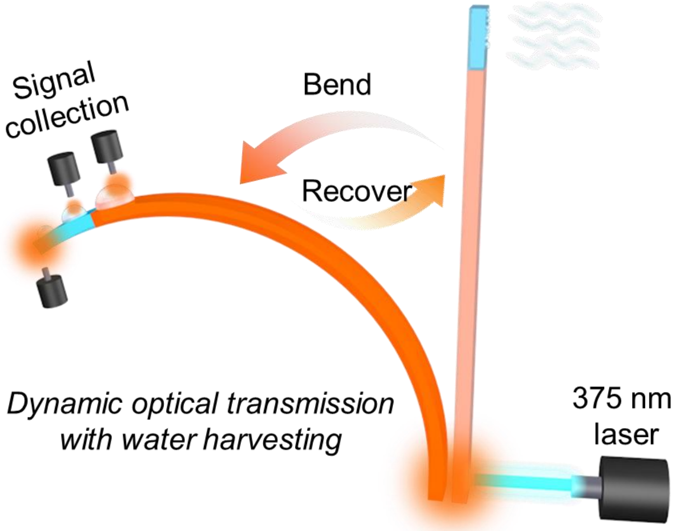

Supplementary Figure 21. Schematic of the dynamic bending-relaxation process and changes in the waveguide signals of (TTP/P^2/**1**)|**1** during fog collection.

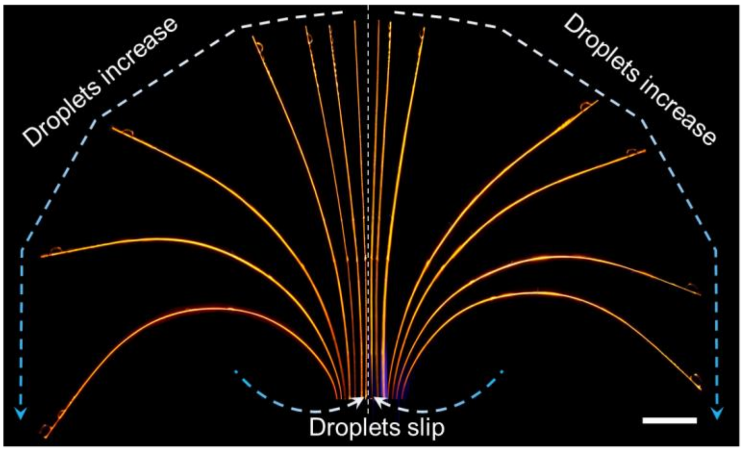

Supplementary Figure 22. Superimposed images showing the stages of the dynamic fog collection by crystal bending of (TTP/P^2/**1**)|**1** (recorded under UV light for enhanced contrast). The hybrid crystal collects droplets of water and gradually bends due to their weight, after which the droplets slide off of the crystal and the crystal straightens up. The scale length is 5 mm

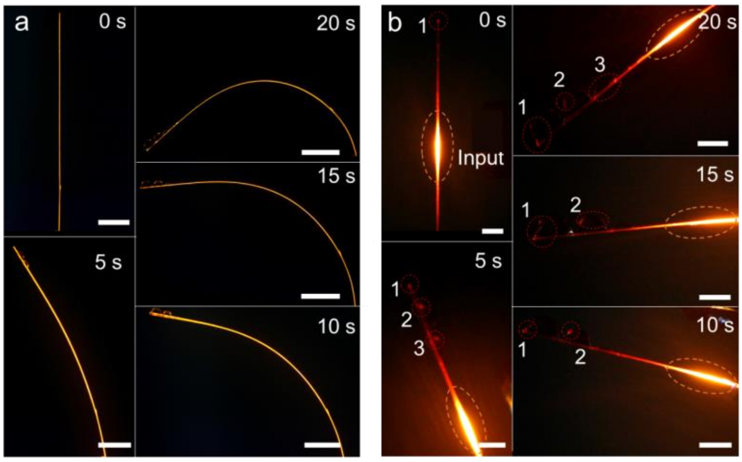

Supplementary Figure 23. Bending recovery of (TTP/P^2/**1**)|**1** during the water harvesting observed under UV light (a) and the corresponding images showing the orientation of the optical waveguide (b) at 0, 5, 10, 15, and 20 s. The scale length in panel a is 5 mm, and in panel b, it is 1 mm.

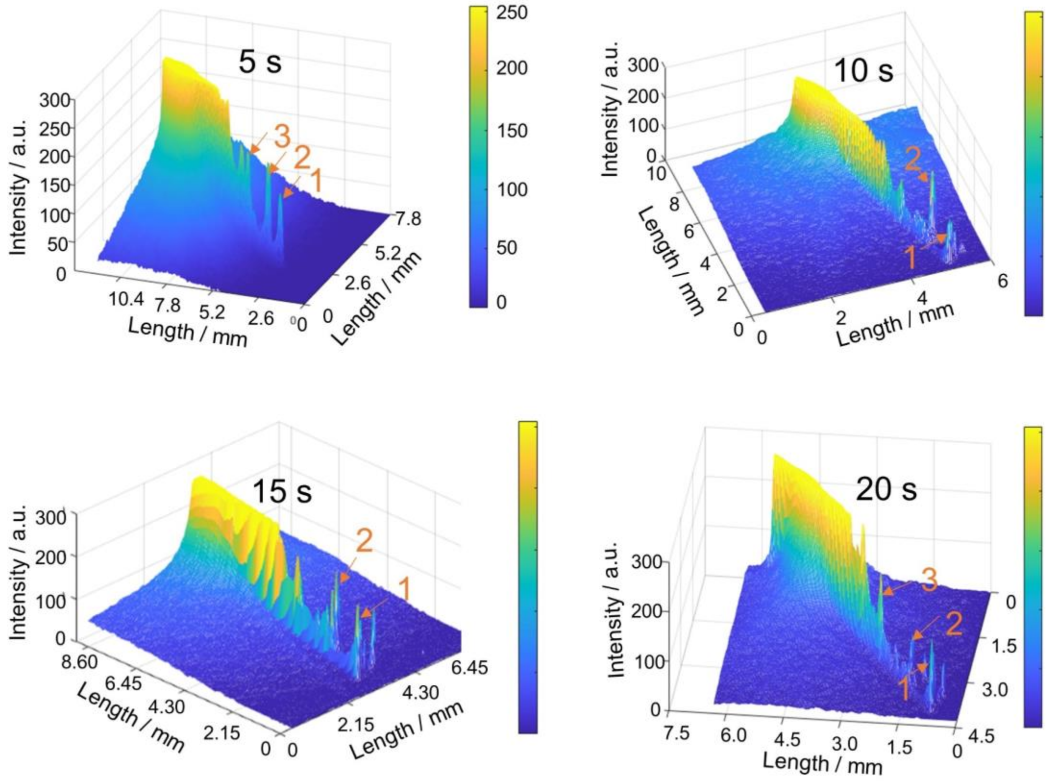

Supplementary Figure 24. Three-dimensional light intensity maps of waveguide signals at 5, 10, 15, and 20 s in panel g of Figure S23. Location 1 represents the output signal at the end of the crystal (which may contain a droplet), and locations 2 and 3 represent the output signal from two droplets.

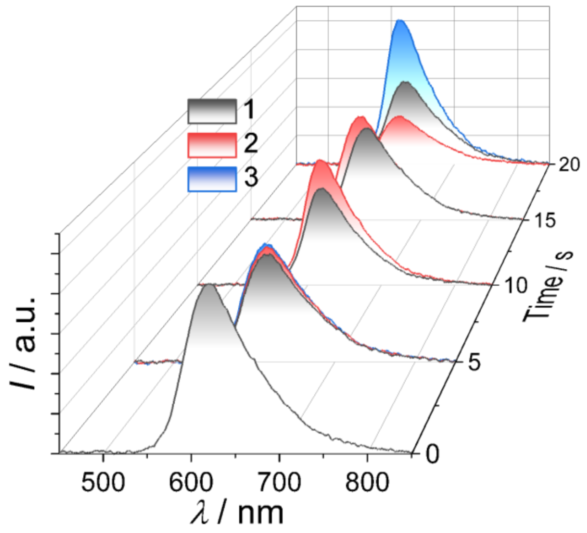

Supplementary Figure 25. Spectra collected at the tip of (TTP/P^2/**1**)|**1** and droplet locations at different time points in the bending during fog collection., and in panel g, it is 1 mm.

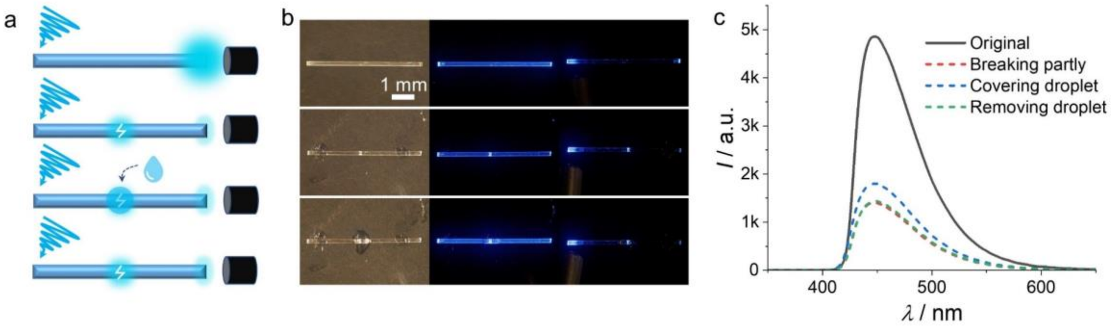

Supplementary Figure 26. (a) Schematic of optical waveguide test of TTP/P^2/**3** under different conditions: The initial crystal is broken in the middle, water droplets are added at the broken location, and finally the droplets are removed. (b, c) The corresponding photographs (b) and emission spectra (c) showing changes in the light signal.

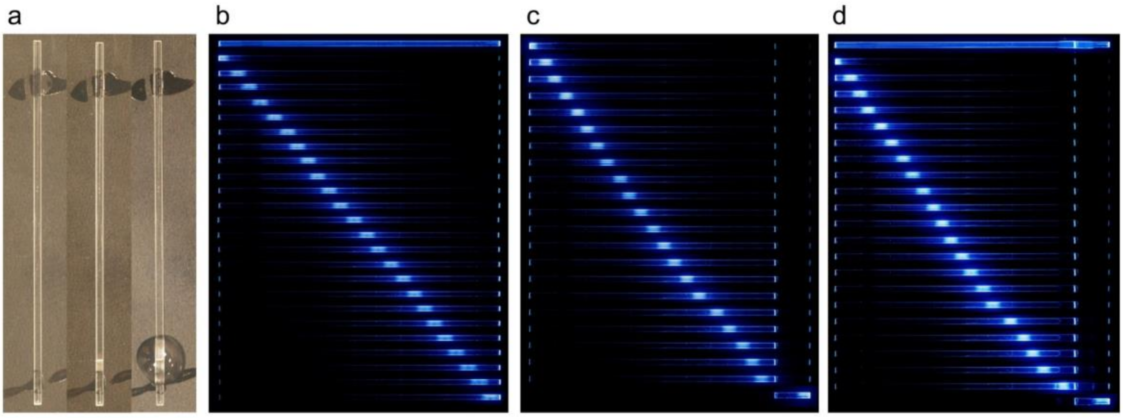

Supplementary Figure 27. (a) Photographs of the TTP/P^2/**3** before and after fracture, as well as the presence of droplets at the fracture site. (b–d) The optical waveguide performance of the original TTP/P^2/**3** (b), broken TTP/P^2/**3** (c) and broken TTP/P^2/**3** with a droplet present at the rupture point (d).

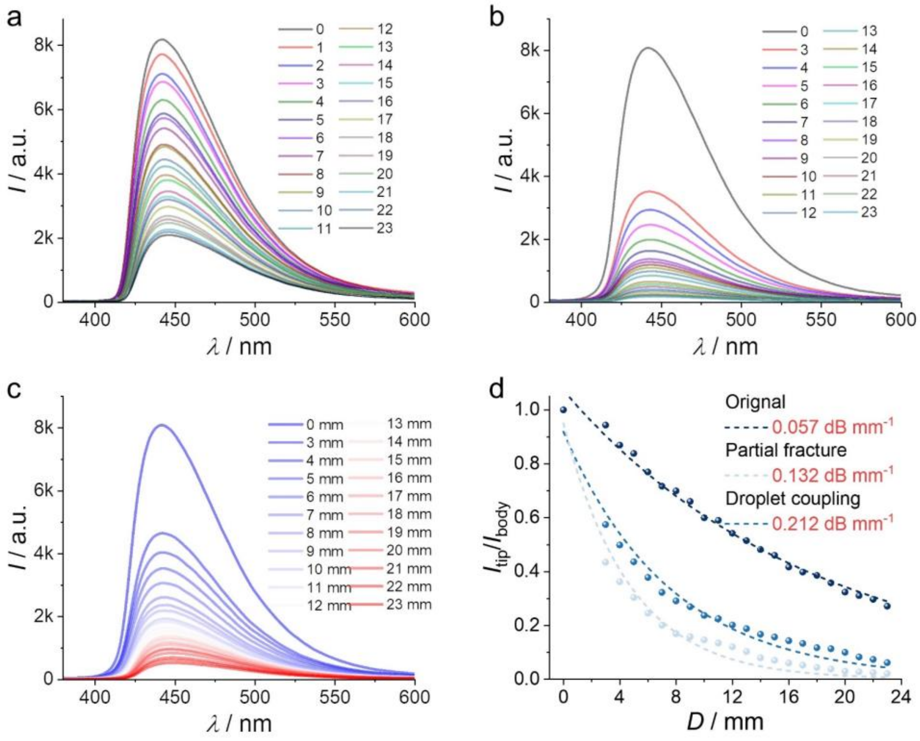

Supplementary Figure 28. (a–c) The corresponding emission spectra collected at one end of the original TTP/P^2/**3** (a), broken TTP/P^2/**3** (b) and broken TTP/P^2/**3** with a droplet present at the rupture point (c). (d) The corresponding $I_{\mathrm{tip}}/I_{\mathrm{body}}$ decays of these crystals. The optical loss coefficients (α) were determined by single-exponential fitting of the function $I_{\mathrm{tip}}/I_{\mathrm{body}} = A\exp(-\alpha D)$, in which I_{tip} and I_{body} are the fluorescence intensities of outcoupled and incidence light respectively, A is the optical loss coefficient, and D is the distance between the excited site and the tip of crystals for collecting emission.

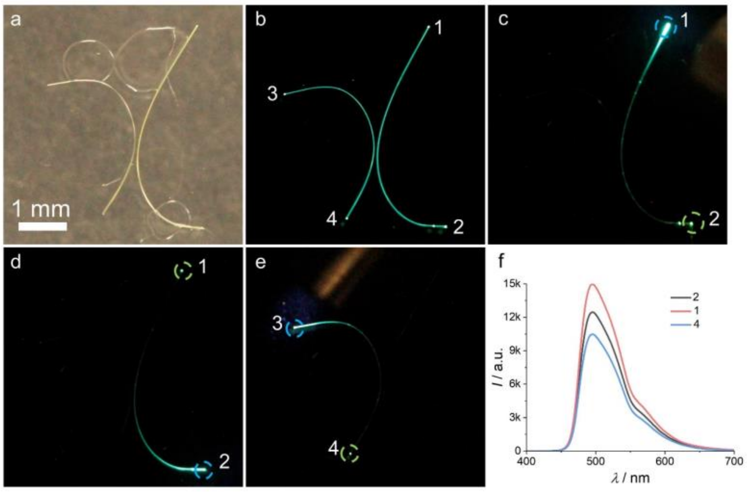

Supplementary Figure 29. (a, b) Photographs of the 2 × 2 coupler consisting of two TTP/P^2/**2** crystals under daylight (a) and UV light (b). (c–e) Fluorescence photographs of terminals 1 (c), 2 (d) and 3 (e) excited by a 355 nm laser. (f) The corresponding waveguide signals for the crystals in panels c–e.

3. Supplementary tables

Supplementary Table 1. The dimensions of crystals **1**–**3** and TTP/P^2/**1**–**3** in three-point bending tests

Crystal	Width / μm	Thickness / μm	Elastic modulus / GPa
**1**	399.3	57.4	6.21
**1**	335.4	59.8	6.44
TTP/P^2/**1**	340.7	51.7	6.10
TTP/P^2/**1**	447.3	54.5	6.13
**2**	935.2	97.5	2.42
**2**	874.5	71.2	2.24
TTP/P^2/**2**	927.6	73.8	1.90
TTP/P^2/**2**	1137	105.2	2.26
**3**	727.9	43.1	2.11
**3**	486.7	65.6	2.16
TTP/P^2/**3**	631.4	44.1	1.86
TTP/P^2/**3**	580.4	60.9	1.92

Supplementary Table 2. Fog collection of Janus crystals (TTP/P^2/**1**–**3**)|**1**–**3**, hybrid crystals TTP/P^2/**1**, **1** and wide lamellar Janus crystal (TTP/P^2/**2**)|**2** in ~95% RH environment (length and width represent the dimensions of the water-collecting part of the crystals)

Sample	Length / mm	Width / μm	Water collection area / cm^2	Collection time / h	Weight of droplets / g	Water collection efficiency / g cm^{-2} h^{-1}
(TTP/P^2/**1**)|**1**	15	246	0.037	1	0.506	13.713
					0.511	13.848
					0.492	13.333
Average: 13.631 ± 0.267 g cm^{-2} h^{-1}						
(TTP/P^2/**2**)|**2**	15	511	0.153	1	0.891	11.647
					0.812	10.621
					0.852	11.124
Average: 11.131 ± 0.513 g cm^{-2} h^{-1}						
(TTP/P^2/**3**)|**3**	15	590	0.177	1	0.872	9.853
					0.898	10.152
					0.951	10.780
Average: 10.262 ± 0.473 g cm^{-2} h^{-1}						
TTP/P^2/**1** (All wrapped)	15	239	0.036	1	0.291	8.083
					0.269	7.472
					0.277	7.694
Average: 7.750 ± 0.309 g cm^{-2} h^{-1}						
**1**	15	385	0.058	1	0.102	1.758
					0.117	2.017
					0.098	1.690
Average: 1.822 ± 0.173 g cm^{-2} h^{-1}						
Wide lamellar (TTP/P^2/**2**)|**2**	15	7148	1.072	1	3.970	3.703
					4.076	3.802
					3.834	3.576
Average: 3.694 ± 0.113 g cm^{-2} h^{-1}						

Supplementary Table 3. Fog collection of (TTP/P^2/**1**)|**1** in different relative humidity environments (the length and width represent the dimensions of the water-collecting part of the crystals)

Sample	Length / mm	Width / µm	Water collection area / cm^2	Relative humidity	Collection time / h	Weight of droplets / g	Water collection efficiency / $g \cdot cm^{-2} \cdot h^{-1}$
(TTP/P^2/**1**)|**1**	15	293	0.044	~95%	1	0.571	12.992
						0.580	13.197
						0.607	13.811
				Average: 13.333 ± 0.426 g cm^{-2} h^{-1}			
				~85%	1	0.294	6.692
						0.315	7.159
						0.276	6.273
				Average: 6.708 ± 0.443 g cm^{-2} h^{-1}			
				~75%	1	0.176	4.010
						0.157	3.568
						0.181	4.114
				Average: 3.897 ± 0.290 g cm^{-2} h^{-1}			

Supplementary Table 4. Fog collection of (TTP/P^2/**1**)|**1** with different width in ~95% RH environment

Length / mm	Width / μm	Water collection area / cm^2	Collection time / h	Weight of droplets / g	Water collection efficiency / g cm^{-2} h^{-1}
15.25	452.58	0.069	1	0.725	10.507
				0.707	10.246
				0.694	10.058
Average: 10.270 ± 0.225 g cm^{-2} h^{-1}					
18.42	369.44	0.068	1	0.704	10.353
				0.735	10.809
				0.818	12.029
Average: 11.064 ± 0.866 g cm^{-2} h^{-1}					
16.58	278.54	0.046	1	0.605	13.152
				0.586	12.739
				0.577	12.543
Average: 12.811 ± 0.311 g cm^{-2} h^{-1}					
14.03	233.23	0.033	1	0.448	13.576
				0.434	13.152
				0.471	14.273
Average: 13.667 ± 0.566 g cm^{-2} h^{-1}					
15.46	175.65	0.027	1	0.388	14.370
				0.401	14.852
				0.372	13.778
Average: 14.333 ± 0.538 g cm^{-2} h^{-1}					
15.74	150.45	0.024	1	0.389	16.208
				0.366	15.250
				0.394	16.426
Average: 15.961 ± 0.626 g cm^{-2} h^{-1}					
14.93	130.84	0.019	1	0.297	15.631
				0.316	16.632
				0.281	14.789
Average: 15.684 ± 0.923 g cm^{-2} h^{-1}					
14.80	112.81	0.017	1	0.253	14.882
				0.234	13.765
				0.260	15.294
Average: 14.647 ± 0.791 g cm^{-2} h^{-1}					
13.75	95.81	0.013	1	0.190	14.615
				0.181	13.923

					0.193	14.846
Average: 14.461 ± 0.480 g cm^{-2} h^{-1}						

Supplementary Table 5. Fog collection of (TTP/P^2/**1–3**)|**1–3** with similar width in ~95% RH environment

Sample	Length / mm	Width / μm	Water collection area / cm^2	Collection time / h	Weight of droplets / g	Water collection efficiency/ g·cm^{-2}·h^{-1}
(TTP/P^2/**1**)|**1**	15	319.65	0.048	1	0.568	11.833
					0.547	11.395
					0.575	11.979
				Average: 11.736 ± 0.304 g cm^{-2} h^{-1}		
(TTP/P^2/**2**)|**2**	15	311.87	0.047	1	0.551	11.723
					0.562	11.957
					0.544	11.574
				Average: 11.751 ± 0.193 g cm^{-2} h^{-1}		
(TTP/P^2/3)|**3**	15	345.14	0.052	1	0.605	11.635
					0.569	10.942
					0.571	10.981
				Average: 11.186 ± 0.389 g cm^{-2} h^{-1}		

Supplementary Table 6. Comparison of water collection rate of the prepared hybrid crystals with other reported works

Water harvesting material	Water collection rate / g cm^{-2} h^{-1}	Experiment details	References
(TTP/P^2/**1**)|**1**<br>(TTP/P^2/**2**)|**2**<br>(TTP/P^2/**3**)|**3**	15.961<br>11.131<br>10.262	The rate of fog flow is ~250 mL / h at 5 cm distance, the temperature and relative humidity (RH) are 20 °C and ~95%	——
A patterned surface with interconnected microchannels (SCL surface)	0.00673	Flow rate of fog stream is ~50 cm / s at ~14.5 cm distance, the temperature and RH are ~20 °C and ~90%	7
TiO_2–Cu composite surface	1.310	A stimulating fog stream is 10 cm / s at ~10 cm distance, the temperature and relative humidity are 20 °C and 80%	8
F-HNT@PDMS coatings	1.058	The water consumption is 150 ± 5 g / h at 5 cm distance	9
A thermodynamically induced interfacial condensation-enhanced fabric (AWF-6)	1.538	The rate of simulated fog flow is 350 mL / h, with 10 cm between the sample and the humidifier nozzle	10
An asymmetric hybrid mesh	2.478	The rate of fog flow is ~300 mL / h at 5 cm distance, the temperature and RH are 20 °C and 90%	11
A superamphiphobic coating with polymer-wrapped particles (SAS surface)	0.8	The rate of a mimetic fog flow is 0.6–0.7 m / s at 18 cm distance	12
A hydrophilic polystyrene flat sheet (CuO-50-PFDT-PS-130)	0.159	The rate of fog flow is ~12 cm / s at 7 cm distance, the temperature and RH are 22 °C and 90–95%	13
CB modified hydrophilic needle and hydrophobic agent coated sheet	1.066	Fog harvesting with a fog flow rate of ~70 cm / s at a 5 cm distance and 85–90 % RH	14
P3 samples	0.203	Fog harvesting with a fog flow rate of ~20 cm/s at a 10	15

		cm distance, the temperature and RH are 24.5 °C and 86%	
Star-shaped wettability patterns	2.78	Fog harvesting with a fog flow rate of ~75 cm / s and RH of more than 95%	16
superhydrophilic (SHL) vein-like pattern on the superhydrophobic (SHB) surface	4.258	Fog harvesting with a fog flow rate of ~1.5 m / s at a 10 cm distance, the temperature and RH are 15 ± 2 °C and 90%	17
Janus mesh membrane (JMM) with an AHS-patterned hydrophobic surface and asymmetric wettability	15.28	Fog harvesting with a fog flow rate of ~20 cm / s at a 10 cm distance, the temperature and RH are 25 °C and 95%	18
Janus fabrics with asymmetric wettability	0.224	Fog harvesting with a fog flow rate of ~10 cm / s at a 10 cm distance, the temperature and RH are 20 °C and 90%	19
PDA coated SU-8 bumps on PPMM surface	0.097	The rate of fog flow is about 10 cm / s at a 10 cm distance, the temperature and RH are 24 °C and 80–85%	20
5-layer deposit patterned strip-groove structural surface	1.61	The rate of fog flow is ~180 mL / h at a 5 cm distance, the temperature is 23 °C	21

Supplementary Table 7. Cyclic stability tests for fog collection of (TTP/P^2/1)|1 in ~95% RH environment for six days.

Sample	Length / mm	Width / µm	Water collection area / cm^2	Collection time / h	Day	Weight of droplets / g	Water collection efficiency/ g cm^{-2} h^{-1}
(TTP/P^2/**1**)|**1**	15	246	0.037	6	1	2.784	14.125
					2	2.702	13.709
					3	2.869	14.556
					4	2.815	14.282
					5	2.730	13.851
					6	2.897	14.698
Average: 14.203 ± 0.387 g cm^{-2} h^{-1}							

Supplementary Table 8. Size and water collection area of the 60 samples (TTP/P^2/**1**)|**1** in the collective fog collection experiment

Width / mm	Length / μm	Water collection area / cm^2	Length / mm	Width / μm	Water collection area / cm^2	Length / mm	Width / μm	Water collection area / cm^2
325.28	18.46	0.074	171.44	18.43	0.039	153.24	19.39	0.037
244.31	16.65	0.05	183.72	14.96	0.034	152.41	19.72	0.037
156.94	18.25	0.035	142.53	15.70	0.028	164.48	15.67	0.032
169.00	16.21	0.034	159.08	13.83	0.027	147.19	20.02	0.036
123.48	18.23	0.028	145.22	15.34	0.027	194.67	16.93	0.041
203.86	13.60	0.034	203.30	17.49	0.044	161.83	17.37	0.035
131.65	15.12	0.025	123.33	19.20	0.029	154.04	19.97	0.038
168.87	15.98	0.033	121.97	13.58	0.020	198.00	16.43	0.040
177.62	14.58	0.032	173.86	18.61	0.040	204.15	17.89	0.045
194.09	17.51	0.042	133.22	17.60	0.029	187.06	15.16	0.035
190.48	18.45	0.043	166.44	18.09	0.037	164.18	18.15	0.037
173.41	15.80	0.034	176.78	17.95	0.039	148.55	18.09	0.033
161.95	18.22	0.036	139.00	18.10	0.031	144.89	19.23	0.034
175.71	14.64	0.032	193.28	16.72	0.040	151.83	17.69	0.033
137.67	17.17	0.029	207.50	14.71	0.038	148.19	18.60	0.034
241.04	19.44	0.058	225.61	15.88	0.044	144.46	16.21	0.029
253.00	14.31	0.045	247.80	17.00	0.052	116.32	18.15	0.026
124.08	18.37	0.028	192.04	15.95	0.038	107.61	16.16	0.021
188.62	15.68	0.037	169.98	16.15	0.034	136.13	19.68	0.033
206.22	19.76	0.050	164.19	14.43	0.029	185.19	15.84	0.036
The total area: 1.76 cm^2								

Supplementary Table 9. The number of droplets, signal intensity and bending angle in each state during the dynamic bending-relaxing process

Time / s	Number of droplets	Bending angle / °	$PL_{input\ 0}$ / a.u.	$PL_{Output\ 1}$ / a.u.	$PL_{Output\ 2}$ / a.u.	$PL_{Output\ 3}$ / a.u.
0 s	0	0	4170	426	—	—
5 s	2	23	4250	312	317	316
10 s	2	53	4396	290	375	—
15 s	3	61	4204	298	335	—
20 s	4	88	4214	288	167	504

4. Legends for the Movies

Supplementary Movie 1. Comparison of the droplet fluidity on the surface of original crystal **1** at 36° tilt and the hybrid crystal TTP/P^2/**1** at 41° tilt.

Supplementary Movie 2. The fog capture and evaporation processes of a crystal **2**. A conventional ultrasonic humidifier with fog-generating power of ~0.25 L h^{-1} was used to evaluate the fog-harvesting performance. The distance between the sample and the outlet was 5 cm. The temperature and relative humidity (RH) were 20 °C and ~95%, respectively.

Supplementary Movie 3. The fog capture and evaporation processes of a hybrid crystal TTP/P^2/**2**. A conventional ultrasonic humidifier with fog-generating power of ~0.25 L h^{-1} was used to evaluate the fog-harvesting performance. The distance between the sample and the outlet was 5 cm. The temperature and relative humidity (RH) were 20 °C and ~95%, respectively.

Supplementary Movie 4. Comparison of fog collection processes of the original crystal **1** and TTP/P^2/**1**. To more clearly reflect the difference in water collection rate, the distance between the fog flow outlet and the crystal was ~1.2 cm.

Supplementary Movie 5. Fog collection with Janus crystals (TTP/P^2/**1**–**3**)|**1**–**3**. To more clearly reflect the difference in water collection rate, the distance between the fog flow outlet and the crystal was ~1.2 cm.

Supplementary Movie 6. The fog collection processes of a wide lamellar Janus crystal (TTP/P^2/**2**)|**2**. To more clearly reflect the difference in water collection rate, the distance between the fog flow outlet and the crystal was ~1.2 cm.

Supplementary Movie 7. The fog collection processes of 60 Janus crystal (TTP/P^2/**1**)|**1**. A conventional ultrasonic humidifier with fog-generating power of ~0.18 L h^{-1} was used to evaluate the fog-harvesting performance. The distance between the sample and the outlet was 9 – 10 cm. The temperature and relative humidity (RH) were 20 °C and ~85%, respectively.

Supplementary Movie 8. Passive optical transduction through a (TTP/P^2/**2**)|**2** during fog collection. A conventional ultrasonic humidifier with fog-generating power of ~0.25 L h^{-1} was used to assess the humidity-harvesting performance. The distance between the samples and the outlet was 5 cm.

Supplementary Movie 9. The dynamic cyclic process in which droplets are collected at the tip of the Janus crystal (TTP/P^2/**2**)|**2**, the crystal bends, and the droplets fall off the crystal.

5. Supplementary references

1. L. Lan, H. Liu, B. Tang, X. Yu, X. Liu, H. Zhang, Polymer-coated organic crystals with solvent-resistant capacity and optical waveguiding function. *Angew. Chem. Int. Ed.* **2021**, *60*, 11283–11287.
2. L. Lan, X. Yang, B. Tang, X. Yu, X. Liu, L. Li, P. Naumov, H. Zhang, Hybrid elastic organic crystals that respond to aerial humidity. *Angew. Chem. Int. Ed.* **2022**, *61*, e202200196.
3. X. Li, L. Chen, D. Cui, W. Jiang, L. Han, N. Niu, Preparation and application of Janus nanoparticles: Recent development and prospects. *Coordin. Chem. Rev.* **2022**, *454*, 214318.
4. C. Yang, N. Han, C. Han, M. Wang, W. Zhang, W. Wang, Z. Zhang, W. Li, X. Zhang, Design of a Janus F-TiO_2@PPS porous membrane with asymmetric wettability for switchable oil/water separation. *ACS Appl. Mater. Interfaces* **2019**, *11*, 22408–22418.
5. L. Yu, G. Y. Chen, H. Xu, X. Liu, Substrate-independent, transparent oil-repellent coatings with self-healing and persistent easy-sliding oil repellency. *ACS Nano* **2016**, *10*, 1076–1085.
6. P. Lyu, X. Zhang, X. Jiang, B. Shang, X. Liu, Z. Deng, One-step preparation of hydrophobic surfaces containing hydrophilic groups for efficient water harvesting. *Langmuir* **2021**, *37*, 9630–9636.
7. Y. Wang, Y. Zhou, P. Han, G. Qi, D. Gao, L. Zhang, C. Wang, J. Che, Y. Wang, S. Tao, Improved water collection from short-term fog on a patterned surface with interconnected microchannels. *Environ. Sci. Technol.* **2024**, *58*, 3812–3822.
8. H. Zhu, Z. Guo, Hybrid engineered materials with high water-collecting efficiency inspired by Namib Desert beetles. *Chem. Commun.* **2016**, *52*, 6809–6812.
9. Y. Hu, M. Zhou, H. Fu, Durable superhydrophobic coating based on halloysite nanotubes for versatile oil/water separation and reusable water collection. *Surf. Interfaces* **2024**, *46*, 104168
10. Y. Ji, W. Yang, X. Li, K. Hou, P. Du, H. Zhao, Z. Fan, B. Xu, Z. Cai, Thermodynamically induced interfacial condensation for efficient fog harvesting. *Small* **2023**, *19*, 2304037.
11. J. Wu, Z. Yan, Y. Yan, C. Li, J. Dai, Beetle-inspired dual-directional Janus pumps with interfacial asymmetric wettability for enhancing fog harvesting. *ACS Appl. Mater. Interfaces* **2022**, *14*, 49338–49351.
12. X. Wang, J. Zeng, X. Yu, Y. Zhang, Superamphiphobic coatings with polymer-wrapped particles: enhancing water harvesting. *J. Mater. Chem. A* **2019**, *7*, 5426–5433.
13. Y. Wang, L. Zhang, J. Wu, N. Mohamed, P. Wang, A facile strategy for the fabrication of a bioinspired hydrophilic–superhydrophobic patterned surface for highly efficient fog-harvesting. *J. Mater. Chem. A* **2015**, *3*, 18963–18969.
14. C. Wen, H. Guo, H. Bai, T. Xu, M. Liu, J. Yang, Y. Zhu, W. Zhao, J. Zhang, M. Cao, L. Zhang, Beetle-inspired hierarchical antibacterial interface for reliable fog harvesting. *ACS Appl. Mater. Interfaces* **2019**, *11*, 34330–34337.
15. H. Park, J. Hwang, T. H. Lee, J. Lee, D. J. Kang, Fog collection based on secondary electrohydrodynamic-induced hybrid structures with anisotropic hydrophilicity. *ACS Appl. Mater. Interfaces* **2021**, *13*, 27575–27585.
16. H. Bai, L. Wang, J. Ju, R. Sun, Y. Zheng, L. Jiang, Efficient water collection on integrative bioinspired surfaces with star-shaped wettability patterns. *Adv. Mater.* **2014**, *26*, 5025–

5030.
17. Q. Wang, F. Yang, Z. Guo, Design and construction of a Laplace and wettability gradient field for efficient water collection. ***Chem. Commun.*** **2023**, ***59***, 6048–6051.
18. S. Song, Y. Zhang, T. Yu, J. Yang, A new Janus mesh membrane with ultrafast directional water transportation and improved fog collection. *J Mater. Sci. Technol.* **2024**, ***202***, 129–139.
19. R. Zhu, M. Liu, Y. Hou, L. Zhang, M. Li, D. Wang, D. Wang, S. Fu, Biomimetic fabrication of Janus fabric with asymmetric wettability for water purification and hydrophobic/hydrophilic patterned surfaces for fog harvesting. *ACS Appl. Mater. Interfaces* **2020**, *12*, 50113–50125.
20. P. Moazzam, H. Tavassoli, A. Razmjou, M. E. Warkiani, M. Asadnia, Mist harvesting using bioinspired polydopamine coating and microfabrication technology. *Desalination* **2018**, *429*, 111–118.
21. Y. Zhang, P. Ming, B. Xue, H. Liu, X. Yang, L. Li, S. Niu, L. Yan, X. Zheng, G. Qin, Facilely fabricating large-area robust heterogeneous wettability surface by mask-patterned ultrafine anode scanning electrodeposition for efficient water collection. *Surf. Interfaces* **2023**, *41*, 103247.
